# Increasing incidence of early-onset type 2 diabetes in Sweden 2006–2021

**DOI:** 10.1093/eurpub/ckaf114

**Published:** 2025-07-09

**Authors:** Sofia Carlsson, Tomas Andersson, Stefan Jansson, Thomas Nyström, Olov Rolandsson, Yuxia Wei

**Affiliations:** Institute of Environmental Medicine, Karolinska Institutet, Stockholm, Sweden; Institute of Environmental Medicine, Karolinska Institutet, Stockholm, Sweden; Faculty of Medicine and Health, Örebro University, University Health Care Research Center, Örebro, Sweden; Department of Clinical Science and Education, Karolinska Institutet, Stockholm, Sweden; Department of Public Health and Clinical Medicine, Family Medicine, Umeå University, Umeå, Sweden; Institute of Environmental Medicine, Karolinska Institutet, Stockholm, Sweden

## Abstract

Our aim was to provide new data on the incidence, prevalence, and secular trend of type 2 diabetes (T2D) in Sweden, specifically early-onset T2D. We followed the Swedish population 2006 to 2021 and calculated age-standardized incidence (per 100 000) and prevalence (%) of T2D (overall) and early-onset T2D (age 23–39 years) stratified by sex, region of birth, and educational level. We projected the future prevalence of early-onset T2D by combining observed trends with population projections. From 2006 to 2021, the prevalence of T2D rose from 4.87% to 7.50%, and incidence from 477 [95% confidence interval (CI) 471–482] to 574 (CI 568–579). Early-onset T2D incidence increased from 54 to 107 (4.7% annual rise; CI 3.7%–5.7%) during this period. Incidence of early-onset T2D was higher in individuals born outside Europe (211, CI 195–226 vs 89, CI 84–93 in 2021) or low education (204, CI 185–223 vs 71, CI 65–77 in 2021), but a rise in incidence was seen irrespective of educational level, region of origin, and sex. If the incidence of early-onset T2D continues to increase at the same pace, its prevalence is projected to increase from 0.64% in 2021 to 3.2% in 2050. While T2D incidence rose marginally in Sweden 2006 to 2021, there was a significant rise in early-onset T2D, seen across different socioeconomic characteristics, with prevalence more than doubling and incidence nearly doubling. This development calls for targeted preventive efforts.

## Introduction

The prevalence of diabetes mellitus in adults has been rising globally over the last decades [1]. The incidence trend is more encouraging, with signs of stable or decreasing incidence in most high-income countries in Europe, including Sweden, and in the USA from the mid-2000s [2]. The rise in prevalence in such countries may be driven primarily by increased life expectancy in people with diabetes, which results in longer duration of diabetes and thus a larger proportion of the population living with the disease [3]. On the other hand, the latest Swedish reports were based on data until 2013, and it is not clear if the decline in incidence has persisted [4, 5].

A concerning development is that type 2 diabetes (T2D) seems to be increasingly diagnosed in adolescents and younger adults [6]. The trend is worrisome, as early-onset T2D appears to be more aggressive, posing a greater risk of complications, comorbidities, and mortality compared to T2D diagnosed at older ages [7–9]. According to the 10th Diabetes Atlas, the global prevalence of T2D among individuals aged 20–39 years, often referred to as ‘early-onset T2D’ rose from 2.9% in 2013–3.8% in 2021 [10]. Based on the Global Burden of Disease study, it was estimated that the incidence of early-onset T2D rose by 56% from 1990 to 2019 [6]. Considerable variation exists within countries, with ethnic minorities and individuals in the lowest socioeconomic strata facing the greatest risk [6, 7].

Research on early-onset T2D in the Nordic countries is limited, but rising incidence was recently reported in Finland and Denmark [11]. In Sweden, no epidemiological data on early-onset T2D have been published. Our aim was to present new data on diabetes trends in Sweden from 2006 to 2021, with a particular focus on early-onset T2D. Additionally, we projected the future burden of early-onset T2D by modelling prevalence, incidence, alongside population projections.

## Methods

### Study population

We included all individuals born between 1895 and 1998 who were living in Sweden in 2006, identified through the Population Register. Everyone was followed for a diabetes diagnosis 2006–2021. Since those born in 1998 were the youngest cohort in the study, they turned age 23 in 2021, making age 23 the youngest age group that could be followed throughout the study period.

### Diabetes

We retrieved information on T2D by combining the National Patient, Diabetes and Prescribed Drug Registers (PDRs). The Patient Register (PR) contains data on all diagnoses, coded according to the Swedish version of the International Classification of Diseases (ICD-10 since 1997), for hospital admissions since 1987 and outpatient specialist care since 2001, but does not include primary care where T2D is mainly treated [12]. The National Diabetes Register (NDR), established in 1996, collects information on diabetes care from Swedish health centres and covers 87% of all adult diabetes patients [13]. The PDR records all filled prescriptions since 2005, using the Anatomical Therapeutic Chemical (ATC) classification system [14]. T2D was defined as (a) a record of T2D in NDR or (b) a record of ICD-10 code E11 in PR or (c) a prescription of metformin (ATC code A10BA02) in PDR, or a prescription of metformin and another glucose-lowering drug (ATC code A10) if it was a woman under age 40 [to avoid including women prescribed metformin due to polycystic ovary syndrome (PCOS)]. The date of diagnosis was defined as the first record in either Register. We defined ‘early onset’ as T2D diagnosed between age 23 and 39. People ever recorded with type 1 diabetes (T1D) or other types of diabetes were excluded from the T2D group.

### Sociodemographic and clinical characteristics

Information on education and country of birth was obtained by linkage to the longitudinal integration database for health insurance and labour market studies (LISA) [15]. Highest recorded education was used to categorize people as having ‘Primary’ (compulsory education 9 years), ‘Secondary’ (upper secondary education 3–4 years), or ‘Tertiary’ (college or university) level education. We classified individuals based on whether they were born within or outside Europe. For people who developed T2D 2006–2021, we retrieved clinical information from NDR from the year of diagnosis or the earliest recording thereafter including smoking, body mass index (kg/m^2^), haemoglobin A1C (HbA1c), blood pressure, triglycerides, estimated glomerular filtration rate (eGFR), and albuminuria (micro and macro albuminuria).

### Statistical analyses

The characteristics of the total population and individuals with T2D were summarized as proportions (%) for categorical variables and means with standard deviations for continuous variables.

Prevalence and incidence rates were calculated annually by sex and 5-year age strata. Rates were directly standardized to the age and sex structure of the 2021 full study population (for prevalence) and the 2021 at-risk population (for incidence). We stratified the analyses by age, sex, education, and country of origin. Stratified analyses were standardized using the same weights. Trends were analysed on the annual standardized estimates using a meta-regression approach with inverse variance weighting, employing an identity link for prevalence and a log link for incidence. This approach implies that prevalence trends represent yearly additive percentage point changes, while incidence trends reflect yearly multiplicative percentage changes.

Time resolution was based on calendar years, with age defined as the difference between the calendar year and the birth year. Individuals were followed from 2006 to 2021, with those who died or emigrated excluded from the study population in subsequent years. For the population at risk, the same criteria were applied, with the additional exclusion of individuals with any diabetes indication in previous years. All specific or aggregated estimates were presented with corresponding 95% confidence intervals (CIs). The analyses were run for T2D diagnosed at any age ≥23 years, for early-onset T2D (age 23–39), and for finer age groups (age 23–29, 30–34, 35–39 years).

Prevalence projections for early-onset T2D were developed using sex- and age-specific prevalence estimates from 2021, combined with age-specific incidence trends to estimate prevalence in subsequent years. These prevalence rates were applied to official national population data for individuals aged 23–39 for the years 2022 and 2023, as well as official population projections for 2024–2050 from Statistics Sweden [16]. The model applied age- and sex-specific survival probabilities from official population projections equally to individuals with and without diabetes. No additional adjustment was made for diabetes-specific excess mortality, as overall mortality in this age group is low and any such excess was considered negligible for the projection.

All analyses were performed using SAS (version 9.4, M8; SAS Institute Inc., Cary, NC, USA).

The sensitivity analyses included (1) assessing the incidence trend of early-onset T2D only using data from NDR since it does not include gestational diabetes and (2) calculating the incidence trend in people aged 40 years or older to see if incidence was only increasing in younger age groups.

The study was approved by the Swedish Ethical Review Authority (2021-02881).

## Results

### Characteristics

The study included 9 193 524 individuals born between 1895 and 1998 and alive in 2006, comprising 4 581 568 (49.8%) males and 4 611 956 (50.2%) females (Supplementary Table S1). Of the population, 82.8% were born in Sweden, 8.4% were born in other European countries, and 8.8% were born outside Europe. There were 848 809 cases of T2D overall, 468 728 (55.2%) in men and 380 081 (44.8%) in women. People with T2D were older and more likely to have shorter education than those without T2D. During the follow-up period 2006–2021, 529 785 incident cases of T2D was recorded overall, together with 24 210 cases of early-onset T2D.

### Overall trend

**Figure 1.**
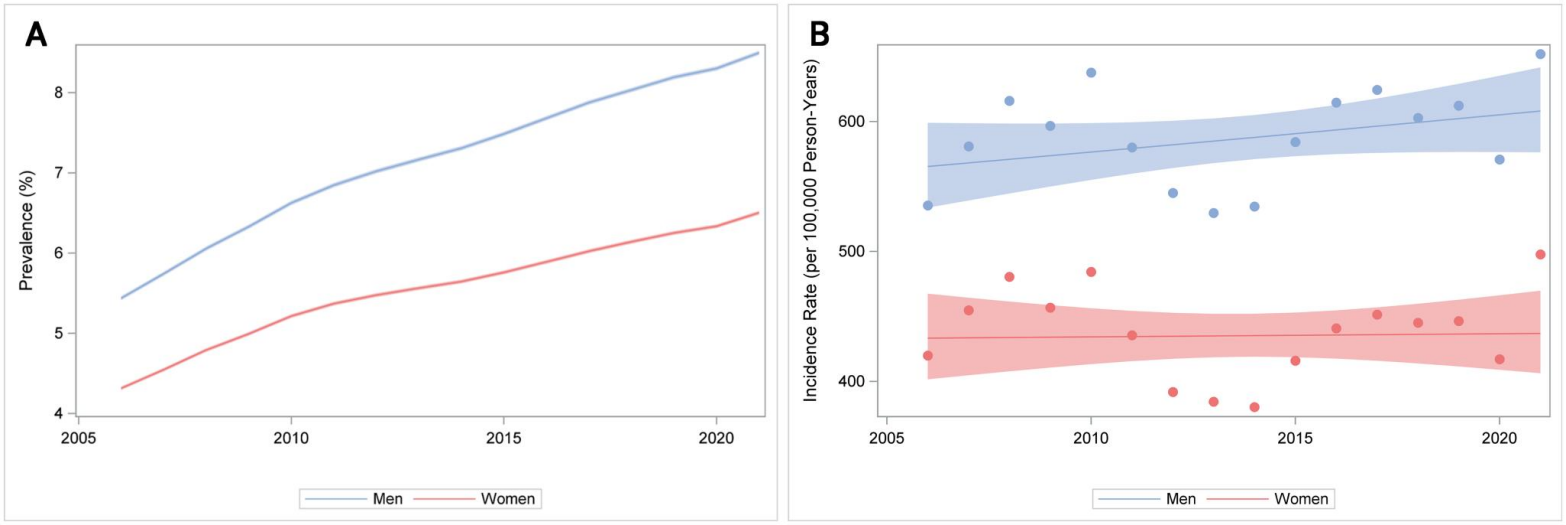
(A) Age-standardized prevalence (%) of type 2 diabetes in Sweden 2006–2021 by sex. The shaded areas represent 95% confidence intervals. (B) Age-standardized incidence (per 100 000 person-years) of type 2 diabetes in Sweden 2006–2021 by sex. The shaded areas represent 95% confidence intervals.

The prevalence of T2D increased from 4.87% to 7.50% between 2006 and 2021; in men, from 5.44% to 8.50%, and in women, from 4.31% to 6.50% (Fig. 1A, Supplementary Table S2). The incidence was estimated at 477 per 100 000 in 2006 and increased slightly to 574 per 100 000 in 2021 (Fig. 1B, Supplementary Table S3). Corresponding estimates for men and women were 535 and 420 in 2006, respectively, and 652 and 498 in 2021. This corresponds to an annual change of 0.3% (95% CI −0.4% – 1.0%) overall, 0.5% (95% CI −0.1% – 1.1%) in men and 0.1 (95% CI −0.8% – 0.9%) in women. When comparing the incidence of T2D over age in 2021 and 2006, we noted a shift towards younger age at diagnosis in 2021 (Supplementary Fig. S1). Both incidence and prevalence of T2D were higher in people born outside of Europe compared to within Europe (Supplementary Tables S4 and S5). Looking at specific regions revealed that incidence was highest in people born in Africa and Asia and lowest in people born in Sweden (Supplementary Fig. S2). We also observed higher T2D incidence and prevalence in people with primary and secondary education vs tertiary education (Supplementary Tables S6 and S7).

### Early-onset T2D

**Figure 2.**
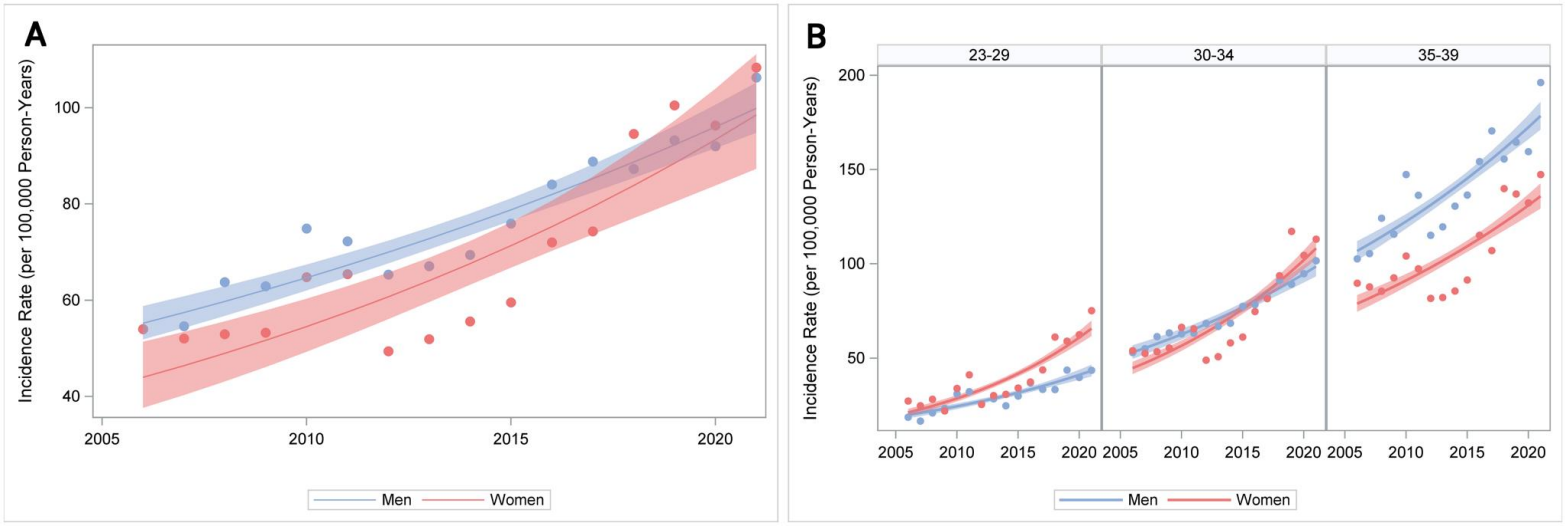
(A) Age-standardized incidence (per 100 000 person-years) of early-onset T2D in Sweden 2006–2021 by sex. Annual change: 4.7% (CI 3.7%–5.7%) overall, 4.0% (CI 3.4%–4.7%) in men, 5.5% (CI 3.8%–7.2%) in women. The shaded areas represent 95% confidence intervals (CI). (B) Incidence (per 100 000 person-years) of early-onset T2D in Sweden 2006–2021 by age and sex. Annual change: age 23–29; 6.7% (CI 6.0%–7.4%) overall, 5.3% (CI 4.4%–6.4%) in men, 7.9% (CI 3.8%–7.2%) in women, age 30–34; 5.1% (CI 4.6%–5.6%) overall, 4.2% (CI 3.5%–4.9%) in men, 6.1% (CI 6.9%–8.8%) in women, age 35–39; 3.6% (CI 3.2%–4.0%) overall, 3.5% (CI 3.0%–4.4%) in men, 3.7% (CI 3.1%–4.9%) in women. The shaded areas represent 95% confidence intervals (CI).

The prevalence of T2D among people aged 23–39 years increased from 0.27% to 0.64% 2006–2021, from 0.25% to 0.58% in men and from 0.29% to 0.70% in women (Supplementary Table S2). During the same period, incidence of early-onset T2D rose from 54 to 107 per 100 000, from 54 to 106 in men and from 54 to 108 in women (Fig. 2A, Supplementary Table S3). The annual rise in early-onset T2D incidence was 4.7% (95% CI 3.7%–5.7%) overall, 4.0% (95% CI 3.4%–4.7%) in men and 5.5% (95% CI 3.8%–7.2%) in women. Stratifying the analyses by finer age groups (23–29 years, 30–34 years and 35–39 years) showed that incidence rose faster in the youngest age group (annual rise 6.7%, CI 6.0–7.4% for age 23–29) than in the oldest age group (annual rise 3.6%, CI 3.2%–4.0% for age 35–39 years) (Fig. 2B). We also found that young (age 23–29) women had higher incidence of T2D than young men and furthermore that the incidence increased more rapidly among younger women than men (Fig. 2B).

**Figure 3.**
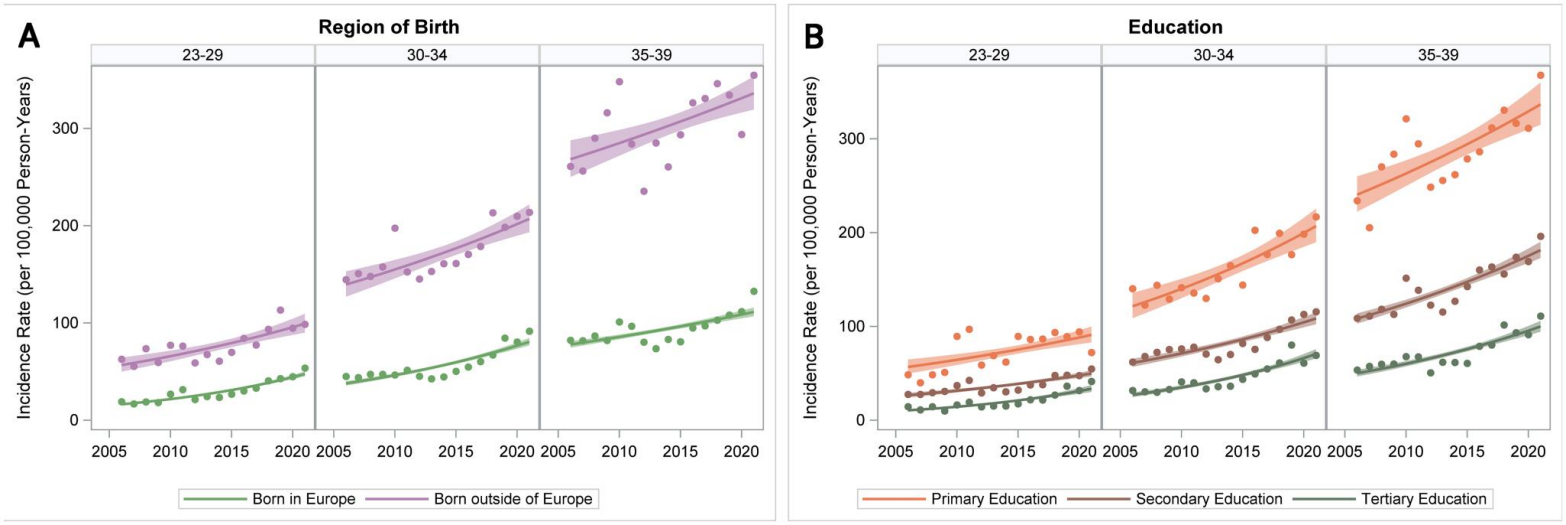
(A) Incidence (per 100 000 person-years) of early-onset T2D 2006–2021 by age and region of birth. The shaded areas represent 95% confidence intervals. Annual change; in people born in Europe: age 23–29; 7.4% (CI 6.6%–8.2%), age 30–34; 5.2% (CI 4.5%–5.8%), age 35–39; 2.4% (CI 1.9%–2.9%). In people born outside Europe: age 23–29, 3.8% (CI 2.5%–5.2%), age 30–34, 3.8% (CI 2.5%–5.2%), age 35–39, 1.5% (CI 0.8%–2.2%). (B) Incidence (per 100 000 person-years) of early-onset T2D 2006–2021 by age and highest attained education. The shaded areas represent 95% confidence intervals. Annual change; in people with primary education: age 23–29; 3.2% (CI 1.9%–4.5%) age 30–34; 3.6% (CI 2.5%–4.8%), age 35–39; 2.3% (CI 1.4%–3.1%). In people with secondary education: age 23–29, 4.3% (CI 3.3%–5.2%), age 30–34, 3.9% (CI 3.1%–4.7%), age 35–39, 3.5% (CI 2.9%–4.1%). In people with tertiary education: age 23–29, 8.1% (CI 6.6%–9.6%), age 30–34, 6.6% (CI 5.6%–7.6%), age 35–39, 4.7% (CI 3.9%–5.5%).

The prevalence of T2D between ages 23 and 39 years was higher in people born outside (1.26%, CI 1.23%–1.30% in 2021) than within Europe (0.47%, CI 0.46%–0.48%) (Supplementary Table S8). Similarly, they had higher incidence of early-onset T2D (2.11, CI 1.95–2.26 per 100 000 in 2021 vs 0.89, CI 0.84–0.93 in those born within vs outside of Europe in 2021) (Fig. 3A, Supplementary Table S9). Incidence increased annually for both groups with a 2.3% increase (CI 1.3%–3.4%) for individuals born outside Europe and a 4.3% increase (2.9%–5.7%) for those born within Europe (Fig. 3A, Supplementary Table S9). This pattern was seen in men as well as women (Supplementary Figs S3 and S4).

In the age group 23–39 years, the highest prevalence of T2D was observed among individuals with primary education (1.31%, CI 1.26%–1.35% in 2021), while those with tertiary education had the lowest prevalence (0.40%, CI 0.38%–0.41% in 2021) (Supplementary Table S10). The incidence of early-onset T2D was also greater in individuals with primary education (204, CI 185–223 per 100 000 in 2021) compared to those with secondary (115, CI 108–122 per 100 000 in 2021) or tertiary education (71, CI 65–77 per 100 000 in 2021) (Fig. 3B, Supplementary Table S11). This pattern was consistent across sex (Supplementary Figs S5–S7). Incidence of early-onset T2D increased from 2006 to 2021 across all educational groups, but the rise was more pronounced among individuals with tertiary compared to primary education, with annual increases of 5.9% (CI 4.5%–7.4%) and 3.0% (CI 2.1%–3.8%), respectively (Fig. 3B, Supplementary Table S11).

### Projection

**Figure 4.**
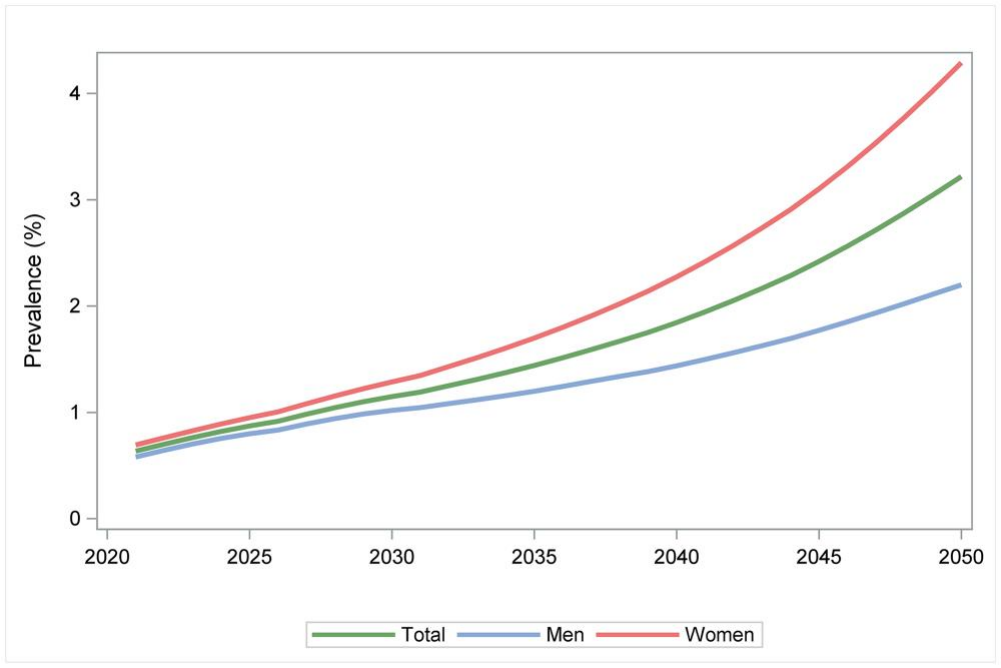
Projected prevalence (%) of type 2 diabetes in the age group 23–39 years, 2022–2050.

With a continued increase in incidence, the prevalence of early-onset T2D is projected to increase in Sweden and reach 3.22% by year 2050; 4.29% in women and 2.20% in men (Fig. 4, Supplementary Table S12).

### Clinical characteristics of T2D by age at diagnosis

People with early-onset T2D were more likely to be obese and smoke (71.6% and 22.4%, respectively) than those diagnosed at age ≥40 (50.3% vs 15.5%) and less likely to have within target levels of HbA1c (50.3% in early-onset T2D vs 64.0% in late-onset T2D) and triglycerides (Supplementary Table S13). Blood pressure and renal function (eGFR) were better in early- vs late-onset T2D. Among early-onset cases, the youngest group (23–29 years) had the highest smoking and obesity rates and the lowest HbA1c target achievement (Supplementary Table S14).

### Sensitivity analyses

When restricting the analyses to early-onset T2D identified through the NDR, there was still an increasing incidence trend (annual increase 2.9%, CI 2.0%–3.8% overall, 2.6%, CI 1.8%–3.4% in men, 3.2%, CI 2.0%–4.4% in women). Restricting the analyses to T2D diagnosed at age ≥40 years also showed a rise in incidence from 677 (CI 675–680) per 100 000 in 2006–1032 (CI 1030–1035) per 100 000 in 2021 (data not shown).

## Discussion

Our nationwide study shows that Sweden experienced a substantial increase in early-onset T2D between 2006 and 2021, with prevalence more than doubling and incidence almost doubling. Incidence and prevalence were highest among individuals born outside Europe and those with lower education levels, yet the increasing trend was observed across these sociodemographic groups as well as in both men and women. If the development continues, we project that the prevalence of T2D among individuals aged 23–39 years will reach 3.2% by year 2050. People with early-onset T2D are more likely to have obesity and poor glycaemic control compared to those diagnosed with T2D at higher ages, which may have severe consequences for their prognosis. The overall prevalence of T2D was estimated at 7.5% in 2021 which is a significant increase from the 4.9% observed in 2006. Overall incidence was also increasing slightly. The rising prevalence of T2D, coupled with the rapid increase in early-onset cases, poses a significant public health threat which will place a growing strain on the healthcare system and society at large.

Our findings align with results based on the Global Burden of Disease Study which showed a clear upward trend in the incidence of early-onset T2D (age 20–39) between 1990 and 2019 across virtually all countries [6]. However, they estimated that the global incidence of early-onset T2D was 183 per 100 000 in 2019 which is 70% higher than the 107 per 100 000 we observed. The discrepancy may partly be due to methodological differences, as their estimate was modelled based on prevalence and mortality rates rather than actual case counts and included undiagnosed diabetes. In a recent analysis of data from Australia, Denmark, Finland, Hungary, Japan, Scotland, South Korea, and Spain covering the period 2000–2020, half of the countries including Finland and Denmark showed increased incidence of T2D in the age group 15–39 years, with annual increases from 2.0 to 8.5% while incidence declined or remained stable in the other countries [11]. Our findings provide further support for an increase in early-onset T2D in the Nordic countries. We can also extend these findings by showing that incidence of early-onset T2D is increasing in the population in Sweden, irrespective of region of origin (Europe vs outside Europe), and educational level, although the rates per se varied greatly across sociodemographic indicators.

We observed a higher incidence of early-onset T2D in Sweden compared to the rates reported from Denmark and Finland [11]. One possible contributing factor is differences in the investigated age range which was 15–39 years in the multi-country study vs 23–39 years in our study. It may also in part be due to Sweden having a larger proportion of immigrants from non-European countries, particularly North Africa and the Middle East than the other Nordic countries [17]. These regions have the highest prevalence of early-onset T2D according to the IDF atlas [17]. In line with this, we note twice as high incidence of early-onset T2D in people born outside Europe compared to within Europe, and incidence was highest in people born in Asia and Africa. Previous studies show that ethnic minorities are disproportionately affected by early-onset T2D [18, 19]. The higher incidence of early-onset T2D in certain ethnic groups may reflect greater genetic susceptibility and increased exposure to lifestyle factors that promote weight gain [7]. Additionally, a larger proportion of individuals immigrating to Sweden from outside Europe have lower education levels, which are linked to a higher risk of early-onset T2D [6, 18]. In our study, individuals with primary education were nearly three times more likely to be diagnosed with T2D before age 40 compared to those with tertiary education. These findings highlight the urgent need for tailored interventions to prevent obesity and subsequent T2D in minority populations.

In general, T2D was more common in men than in women, confirming previous observations [10]. However, young women (age 23–29) had higher incidence than men. The reason could be that misclassification of gestational diabetes inflated our incidence estimates in women. It is also possible that routine screening for gestational diabetes during pregnancy may lead to earlier detection of T2D in women. Our findings align with results based on a national diabetes audit in England showing that T2D in women predominate up until the age of 25, after which men become the more affected sex [18]. One explanation could be that young women have additional risk factors for T2D such as gestational diabetes and PCOS, which may make them more susceptible to early-onset T2D than men. The rise in females is concerning considering that maternal diabetes is linked to adverse pregnancy outcomes and negative long-term health effects in the offspring [20, 21].

Regarding the overall trend, we observed a slight increase in T2D incidence also among individuals over 40, signalling a reversal of the declining trend previously observed in Sweden until 2013 [4, 5]. Magliano *et al.* reported stable or declining diabetes/T2D incidence between 2006 and 2014 in two-thirds of 47 reviewed studies including predominantly high-income populations [2]. Declines in T2D incidence have been observed also after 2015 in some European countries, including the Netherlands, France, and Germany [22–24]. It is possible the pandemic contributed to the rise seen in Sweden, since COVID-19 infection has been linked to higher T2D incidence, and we observed the highest T2D incidence in 2021 [25]. This does not apply to early-onset T2D however, since its incidence rose consistently from 2006 to 2021. Additionally, we noted a concerning shift towards younger ages at T2D diagnosis, which implies a longer disease duration and an associated increase in the risk of complications.

Strengths of the study include using nationwide data with access to information on diabetes, sociodemographic factors, and, for people with diabetes, clinical characteristics. To ensure accurate identification and timing of T2D, we combined three high quality registers. A limitation was the inability to assess trends among individuals below the age of 23, as this was the youngest age group for which data were available through 2021. We also lacked information on parental country of origin, which meant we could not assess incidence of T2D separately in those with immigrant parents. Another concern is the classification of diabetes type. To avoid T1D, we only included people recorded as having T2D and excluded anyone ever recorded with a diagnosis of T1D. Importantly, we recently showed that the incidence of adult-onset T1D (age 19–30) increased by 6% per 5 years in Sweden between 2001 and 2020, whereas we observe a 23.5% increase in early-onset T2D per 5 years [27]. It is possible that some women had gestational diabetes instead of T2D. However, it is important to note that NDR does not include gestational diabetes, and a rising trend was observed when our incidence estimates were based solely on this register. We applied stricter criteria for defining early-onset T2D in women and required that women identified through PDR had another glucose-lowering drug in addition to metformin. This ensured exclusion of women using metformin solely on the indication PCOS. This may have led us to underestimate the occurrence of early-onset T2D in women.

Although early-onset T2D remains uncommon, its prevalence is rising rapidly, making up an increasing share of total T2D cases. According to our findings, early-onset T2D represented 10% of all new T2D cases in 2006, increasing to 16% by 2021. This is alarming trend since earlier T2D diagnosis is associated with a higher risk of a range of macrovascular outcomes and shorter life expectancy that T2D diagnosed later [8, 9]. One reason may be that earlier onset leads to longer exposure to hyperglycaemia. Additionally, T2D at younger ages seems to have a more aggressive phenotype leading to faster disease progression and poorer glycaemic control [26]. In support hereof, we find that those with early onset T2D have higher HbA1c levels than those diagnosed later, especially younger men. More than 70% of men and women with early-onset T2D were obese which also puts them at increased risk of cardiovascular complications [28].

The rise of early-onset T2D in Sweden signals an emerging threat to public health, as these patients have a high risk of complications and may face shortened life expectancy. As early-onset T2D affects individuals during their prime working, childbearing, and child-raising years, its impact is likely to be more far-reaching than T2D diagnosed later in life. It disproportionally affects individuals from minority ethnic backgrounds, those with lower levels of education and young women, in childbearing ages, with potential implications also for pregnancy outcomes. This highlights the need for targeted primary and secondary prevention to reduce the T2D risk in the population and improve prognosis of those affected. Further evidence on potential risk factors is needed to develop new preventive strategies to address the rising incidence of T2D in younger individuals.

## Supplementary Material

ckaf114_Supplementary_Data

## Data Availability

Our data are register-based data that are pseudonymized and thus subject to GDPR and cannot be shared openly. Only metadata is published openly. Underlying data from the registers can be made available upon request to rdo@ki.se after ensuring compliance with relevant legislation and GDPR. Key points Early-onset type 2 diabetes incidence in Sweden nearly doubled from 2006 to 2021. Individuals with low education, those born outside Europe and young women are disproportionally affected. Prevalence of early-onset T2D is projected to reach 3.2% in Sweden by 2050.
