# Supplementary material for: Increasing incidence of early-onset type 2 diabetes in Sweden 2006–2021
Source: Eur J Public Health. 2025 Jul 9;35(6):1258–63. doi: 10.1093/eurpub/ckaf114 (PMC12707497; doi:10.1093/eurpub/ckaf114)
Supplement: ckaf114_Supplementary_Data [file ckaf114_supplementary_data.pdf]

# Supplementary material

Table S1. Characteristics of the Swedish population born 1895-1998, followed 2006-2021

Table S2. Age-standardized prevalence (%) with 95% confidence intervals of type 2 diabetes and early-onset T2D 2006 to 2021, overall and by sex.

Table S3. Age-standardized incidence (per 100 000) with 95% confidence intervals of type 2 diabetes and early-onset T2D 2006 to 2021, overall and by sex.

Table S4. Age-standardized prevalence (%) with 95% confidence intervals of type 2 diabetes 2006 to 2021, by birth region (Europe vs. outside Europe)

Table S5. Age-standardized incidence (per 100 000) with 95% confidence intervals of type 2 diabetes 2006 to 2021, by birth region (Europe vs. outside Europe).

Figure S1. Incidence (per 100 000) of type 2 diabetes 2006 and 2021by age in men and women.

Figure S2. Age-standardized incidence (per 100 000) with 95% confidence intervals of type 2 diabetes 2006 to 2021 by birth region.

Table S6. Age-standardized prevalence (%) with 95% confidence intervals of type 2 diabetes 2006 to 2021, by educational level.

Table S7. Age-standardized incidence (per 100 000) with 95% confidence intervals of type 2 diabetes 2006 to 2021, by educational level.

Table S8. Age-standardized prevalence (%) of early-onset type 2 diabetes 2006 to 2021, by birth region (Europe vs. outside Europe).

Table S9. Age-standardized incidence (per 100 000) with 95% confidence intervals of early-onset type 2 diabetes 2006 to 2021, by birth region (Europe vs. outside Europe).



For Review Only

Figure S3. Incidence (per 100 000) with 95% confidence intervals of early-onset type 2 diabetes 2006 to 2021by sex in people born within Europe.

Figure S4. Incidence (per 100 000) with 95% confidence intervals of early-onset type 2 diabetes 2006 to 2021by sex in people born outside of Europe.

Table S10. Age-standardized prevalence (%) with 95% confidence intervals of early-onset type 2 diabetes 2006 to 2021, by educational level.

Table S11. Age-standardized incidence (per 100 000) with 95% confidence intervals of early-onset type 2 diabetes 2006 to 2021, by educational level.

Figure S5. Incidence (per 100 000) with 95% confidence intervals of early-onset type 2 diabetes 2006 to 2021by sex in people with primary education.

Figure S6. Incidence (per 100 000) with 95% confidence intervals of early-onset type 2 diabetes 2006 to 2021by sex in people with secondary education.

Figure S7. Incidence (per 100 000) with 95% confidence intervals of early-onset type 2 diabetes 2006 to 2021by sex in people with tertiary education.

Table S12. Projected prevalence of type 2 diabetes in the age group 23-39 year, 2022 to 2050.

Table S13. Clinical characteristics of people with incident type 2 diabetes by age at diagnosis.

Table S14. Clinical characteristics of people with incident early-onset type 2 diabetes by age at diagnosis.

For Review Only

**Table S1. Characteristics of the Swedish population born 1895-1998, followed 2006-2021**

| | Total population | | | People recorded with type 2 diabetes | | |
|---|---|---|---|---|---|---|
| | Total | Men | Women | Total | Men | Women |
| **Population No (%)** | 9 193 524 | 4 581 568 (49.8) | 4 611 956 (50.2) | 848 809 | 468 728 (55.2) | 380 081 (44.8) |
| **Birth year** | | | | | | |
| **1895-1929** | 733 884 (8.0) | 282 673 (6.2) | 451 211 (9.8) | 126 257 (14.9) | 52 869 (11.3) | 73 388 (19.3) |
| **1930-1939** | 749 446 (8.2) | 354 710 (7.7) | 394 736 (8.6) | 163 914 (19.3) | 86 033 (18.4) | 77 881 (20.5) |
| **1940-1949** | 1 192 124 (13.0) | 599 113 (13.1) | 593 011 (12.9) | 229 704 (27.1) | 135 547 (28.9) | 94 157 (24.8) |
| **1950-1959** | 1 206 043 (13.1) | 611 190 (13.3) | 594 853 (12.9) | 163 910 (19.3) | 99 648 (21.3) | 64 262 (16.9) |
| **1960-1969** | 1 361 815 (14.8) | 697 905 (15.2) | 663 910 (14.4) | 101 062 (11.9) | 60 813 (13.0) | 40 249 (10.6) |
| **1970-1979** | 1 372 035 (14.9) | 704 952 (15.4) | 667 083 (14.5) | 43 400 (5.1) | 23 888 (5.1) | 19 512 (5.1) |
| **1980-1989** | 1 419 002 (15.4) | 731 337 (16.0) | 687 665 (14.9) | 16 022 (1.9) | 7 830 (1.7) | 8 192 (2.2) |
| **1990-1998** | 1 159 175 (12.6) | 599 688 (13.1) | 559 487 (12.1) | 4 540 (0.5) | 2 100 (0.4) | 2 440 (0.6) |
| **Education** | | | | | | |
| **Primary** | 1 894 555 (20.6) | 970 627 (21.2) | 923 928 (20.0) | 294 296 (34.7) | 157 028 (33.5) | 137 268 (36.1) |
| **Secondary** | 3 801 164 (41.3) | 2 000 086 (43.7) | 1 801 078 (39.1) | 343 880 (40.5) | 196 706 (42.0) | 147 174 (38.7) |
| **Tertiery** | 3 045 829 (33.1) | 1 384 020 (30.2) | 1 661 809 (36.0) | 162 392 (19.1) | 93 598 (20.0) | 68 794 (18.1) |
| **Missing** | 451 976 (4.9) | 226 835 (5.0) | 225 141 (4.9) | 48 241 (5.7) | 21 396 (4.6) | 26 845 (7.1) |
| **Birth region** | | | | | | |
| **Sweden** | 7 609 060 (82.8) | 3 796 374 (82.9) | 3 812 686 (82.7) | 693 323 (81.7) | 385 923 (82.3) | 307 400 (80.9) |
| **The Nordic countries*** | 231 628 (2.5) | 98 784 (2.2) | 132 844 (2.9) | 31 078 (3.7) | 15 446 (3.3) | 15 632 (4.1) |
| **Europe**** | 543 191 (5.9) | 273 022 (6.0) | 270 169 (5.9) | 50 234 (5.9) | 26 863 (5.7) | 23 371 (6.1) |
| **Asia** | 540 885 (5.9) | 274 110 (6.0) | 266 775 (5.8) | 51 424 (6.1) | 28 120 (6.0) | 23 304 (6.1) |
| **Africa** | 159 662 (1.7) | 85 473 (1.9) | 74 189 (1.6) | 14 481 (1.7) | 8 259 (1.8) | 6 222 (1.6) |
| **South America** | 65 688 (0.7) | 31 656 (0.7) | 34 032 (0.7) | 5 582 (0.7) | 2 781 (0.6) | 2 801 (0.7) |
| **Other** | 43 410 (0.5) | 22 149 (0.5) | 21 261 (0.5) | 2 687 (0.3) | 1 336 (0.3) | 1 351 (0.4) |

*The Nordic countries except Sweden. **Europe except the Nordic countries.

**Table S2. Age-standardized prevalence (%) with 95% confidence intervals of type 2 diabetes and early-onset T2D 2006 to 2021, overall and by sex.**

| | Overall Prevalence (95% CI) | | | Early-onset type 2 diabetes Prevalence (95% CI) | | |
|---|---|---|---|---|---|---|
| **year** | Men | Women | Total | Men | Women | Total |
| **2006** | 5.44 (5.41—5.46) | 4.31 (4.29—4.34) | 4.87 (4.86—4.89) | 0.25 (0.24—0.26) | 0.29 (0.27—0.30) | 0.27 (0.26—0.27) |
| **2007** | 5.74 (5.72—5.77) | 4.55 (4.52—4.57) | 5.14 (5.13—5.16) | 0.26 (0.25—0.27) | 0.30 (0.29—0.31) | 0.28 (0.27—0.29) |
| **2008** | 6.06 (6.03—6.08) | 4.79 (4.77—4.81) | 5.42 (5.40—5.44) | 0.28 (0.27—0.29) | 0.32 (0.31—0.33) | 0.30 (0.29—0.31) |
| **2009** | 6.33 (6.30—6.36) | 4.99 (4.97—5.02) | 5.66 (5.64—5.68) | 0.29 (0.28—0.31) | 0.34 (0.33—0.35) | 0.32 (0.31—0.32) |
| **2010** | 6.63 (6.60—6.65) | 5.21 (5.19—5.24) | 5.92 (5.90—5.94) | 0.32 (0.31—0.33) | 0.37 (0.35—0.38) | 0.34 (0.34—0.35) |
| **2011** | 6.85 (6.82—6.87) | 5.37 (5.35—5.39) | 6.11 (6.09—6.12) | 0.35 (0.34—0.36) | 0.39 (0.38—0.40) | 0.37 (0.36—0.38) |
| **2012** | 7.02 (6.99—7.05) | 5.48 (5.45—5.50) | 6.25 (6.23—6.26) | 0.36 (0.35—0.37) | 0.40 (0.39—0.41) | 0.38 (0.37—0.39) |
| **2013** | 7.17 (7.14—7.19) | 5.56 (5.54—5.59) | 6.36 (6.34—6.38) | 0.38 (0.37—0.39) | 0.41 (0.40—0.42) | 0.39 (0.39—0.40) |
| **2014** | 7.31 (7.28—7.33) | 5.65 (5.62—5.67) | 6.47 (6.46—6.49) | 0.40 (0.38—0.41) | 0.42 (0.41—0.43) | 0.41 (0.40—0.42) |
| **2015** | 7.49 (7.46—7.51) | 5.76 (5.74—5.78) | 6.62 (6.60—6.64) | 0.41 (0.40—0.43) | 0.44 (0.42—0.45) | 0.43 (0.42—0.43) |
| **2016** | 7.68 (7.65—7.71) | 5.89 (5.87—5.91) | 6.78 (6.77—6.80) | 0.44 (0.43—0.45) | 0.46 (0.45—0.48) | 0.45 (0.44—0.46) |
| **2017** | 7.88 (7.85—7.90) | 6.02 (6.00—6.05) | 6.95 (6.93—6.97) | 0.47 (0.46—0.48) | 0.50 (0.48—0.51) | 0.48 (0.47—0.49) |
| **2018** | 8.03 (8.01—8.06) | 6.14 (6.12—6.17) | 7.08 (7.07—7.10) | 0.50 (0.48—0.51) | 0.55 (0.53—0.56) | 0.52 (0.51—0.53) |
| **2019** | 8.19 (8.17—8.22) | 6.25 (6.23—6.28) | 7.22 (7.20—7.24) | 0.53 (0.51—0.54) | 0.60 (0.59—0.62) | 0.56 (0.55—0.57) |

**Table S3. Age-standardized incidence (per 100 000) with 95% confidence intervals of type 2 diabetes and early-onset T2D 2006 to 2021, overall and by sex.**

| | Overall Incidence (95% CI) | | | Early-onset type 2 diabetes incidence (95% CI) | | |
|---|---|---|---|---|---|---|
| **Year** | Men | Women | Total | Men | Women | Total |
| **2006** | 535 (527—544) | 420 (413—427) | 477 (471—482) | 54 (50—58) | 54 (49—59) | 54 (51—57) |
| **2007** | 581 (572—590) | 455 (447—462) | 517 (511—523) | 55 (50—59) | 52 (48—57) | 53 (50—57) |
| **2008** | 616 (607—625) | 480 (473—488) | 547 (541—553) | 64 (59—69) | 53 (48—57) | 59 (55—62) |
| **2009** | 597 (588—605) | 457 (449—464) | 526 (520—531) | 63 (58—68) | 53 (49—58) | 58 (55—62) |
| **2010** | 638 (629—647) | 484 (477—492) | 560 (554—566) | 75 (70—80) | 65 (60—70) | 70 (66—74) |
| **2011** | 580 (572—589) | 436 (428—443) | 507 (501—512) | 72 (67—77) | 65 (60—70) | 69 (65—73) |
| **2012** | 545 (537—553) | 392 (385—399) | 467 (462—473) | 65 (60—70) | 49 (45—54) | 58 (54—61) |
| **2013** | 530 (522—538) | 384 (378—391) | 456 (451—461) | 67 (62—72) | 52 (47—56) | 60 (56—63) |
| **2014** | 535 (527—543) | 380 (374—387) | 456 (451—461) | 69 (64—74) | 56 (51—60) | 63 (59—66) |
| **2015** | 584 (576—593) | 416 (409—423) | 499 (493—504) | 76 (71—81) | 60 (55—64) | 68 (64—72) |
| **2016** | 615 (606—623) | 441 (434—448) | 526 (521—532) | 84 (79—89) | 72 (67—77) | 78 (74—82) |
| **2017** | 624 (616—633) | 452 (444—459) | 537 (531—542) | 89 (83—94) | 74 (69—80) | 82 (78—86) |
| **2018** | 603 (594—611) | 445 (438—452) | 523 (517—528) | 87 (82—93) | 95 (89—100) | 91 (87—95) |
| **2019** | 612 (604—620) | 447 (440—454) | 528 (523—534) | 93 (88—99) | 101 (94—107) | 97 (93—101) |
| **2020** | 571 (563—579) | 417 (410—424) | 493 (488—498) | 92 (86—98) | 96 (90—102) | 94 (90—98) |
| **2021** | 652 (643—660) | 498 (490—505) | 574 (568—579) | 106 (100—112) | 108 (102—115) | 107 (103—112) |
| **Annual increase, % (95% CI)** | 0.5 (-0.1—1.1) | 0.1 (-0.8—0.9) | 0.3 (-0.4—1.0) | 4.0 (3.4—4.7) | 5.5 (3.8—7.2) | 4.7 (3.7—5.7) |
| **2020** | 8.30 (8.28—8.33) | 6.34 (6.31—6.36) | 7.32 (7.30—7.33) | 0.55 (0.53—0.56) | 0.64 (0.63—0.66) | 0.59 (0.58—0.60) |
| **2021** | 8.50 (8.47—8.52) | 6.50 (6.48—6.53) | 7.50 (7.48—7.51) | 0.58 (0.57—0.60) | 0.70 (0.68—0.71) | 0.64 (0.63—0.65) |

**Table S4. Age-standardized prevalence (%) with 95% confidence intervals of tpe 2 diabetes 2006 to 2021, by birth region (Europe vs. outside Europe).**

| | Men | | Women | | Total | |
|---|---|---|---|---|---|---|
| **Year** | Born in Europe | Born outside Europe | Born in Europe | Born outside Europe | Born in Europe | Born outside Europe |
| **2006** | 5.34 (5.32—5.37) | 7.93 (7.55—8.30) | 4.22 (4.20—4.24) | 7.28 (6.98—7.57) | 4.78 (4.76—4.80) | 7.60 (7.36—7.84) |
| **2007** | 5.63 (5.60—5.65) | 8.64 (8.30—8.98) | 4.44 (4.42—4.46) | 7.79 (7.50—8.07) | 5.03 (5.02—5.05) | 8.21 (7.99—8.43) |
| **2008** | 5.92 (5.89—5.95) | 9.35 (9.05—9.65) | 4.67 (4.64—4.69) | 8.53 (8.26—8.81) | 5.29 (5.27—5.31) | 8.94 (8.74—9.15) |
| **2009** | 6.17 (6.15—6.20) | 9.99 (9.71—10.27) | 4.85 (4.83—4.87) | 9.12 (8.86—9.39) | 5.51 (5.49—5.53) | 9.55 (9.36—9.75) |
| **2010** | 6.44 (6.42—6.47) | 10.60 (10.33—10.86) | 5.05 (5.03—5.07) | 9.74 (9.48—9.99) | 5.74 (5.73—5.76) | 10.17 (9.98—10.35) |
| **2011** | 6.64 (6.62—6.67) | 11.12 (10.87—11.37) | 5.18 (5.16—5.21) | 10.15 (9.91—10.39) | 5.91 (5.89—5.93) | 10.63 (10.46—10.81) |
| **2012** | 6.79 (6.77—6.82) | 11.54 (11.31—11.78) | 5.27 (5.24—5.29) | 10.48 (10.26—10.71) | 6.03 (6.01—6.05) | 11.01 (10.85—11.18) |
| **2013** | 6.91 (6.88—6.94) | 11.95 (11.73—12.17) | 5.33 (5.31—5.35) | 10.84 (10.62—11.05) | 6.12 (6.10—6.13) | 11.39 (11.24—11.55) |
| **2014** | 7.02 (6.99—7.04) | 12.31 (12.10—12.51) | 5.39 (5.36—5.41) | 11.17 (10.96—11.38) | 6.20 (6.18—6.22) | 11.74 (11.59—11.88) |
| **2015** | 7.16 (7.13—7.19) | 12.67 (12.48—12.87) | 5.47 (5.45—5.49) | 11.50 (11.30—11.69) | 6.31 (6.29—6.33) | 12.08 (11.94—12.22) |
| **2016** | 7.32 (7.29—7.34) | 13.03 (12.85—13.22) | 5.57 (5.54—5.59) | 11.76 (11.57—11.95) | 6.44 (6.42—6.46) | 12.40 (12.26—12.53) |
| **2017** | 7.48 (7.45—7.50) | 13.51 (13.34—13.69) | 5.66 (5.64—5.69) | 12.15 (11.97—12.34) | 6.57 (6.55—6.58) | 12.83 (12.70—12.96) |
| **2018** | 7.60 (7.58—7.63) | 13.83 (13.66—14.00) | 5.75 (5.73—5.77) | 12.44 (12.26—12.62) | 6.67 (6.66—6.69) | 13.13 (13.01—13.26) |
| **2019** | 7.73 (7.71—7.76) | 14.13 (13.96—14.29) | 5.83 (5.81—5.85) | 12.69 (12.52—12.86) | 6.78 (6.76—6.80) | 13.41 (13.29—13.53) |
| **2020** | 7.81 (7.78—7.84) | 14.38 (14.23—14.54) | 5.89 (5.86—5.91) | 12.87 (12.71—13.04) | 6.84 (6.83—6.86) | 13.63 (13.51—13.74) |

**Table S5. Age-standardized incidence (per 100 000) with 95% confidence intervals of type 2 diabetes 2006 to 2021, by birth region (Europe vs. outside Europe).**

| | Men | | Women | | Total | |
|---|---|---|---|---|---|---|
| **year** | Born in Europe | Born outside Europe | Born in Europe | Born outside Europe | Born in Europe | Born outside Europe |
| **2006** | 519 (511—527) | 917 (823—1011) | 408 (400—415) | 846 (727—966) | 462 (457—468) | 881 (805—958) |
| **2007** | 564 (555—573) | 973 (883—1064) | 441 (433—448) | 804 (725—883) | 501 (496—507) | 887 (827—947) |
| **2008** | 595 (586—604) | 1126 (1005—1248) | 463 (456—471) | 987 (884—1091) | 528 (523—534) | 1056 (976—1135) |
| **2009** | 576 (567—584) | 1044 (957—1131) | 438 (431—445) | 958 (864—1051) | 506 (500—512) | 1000 (936—1064) |
| **2010** | 613 (604—622) | 1088 (1002—1174) | 462 (455—470) | 944 (864—1025) | 537 (531—542) | 1015 (956—1074) |
| **2011** | 558 (550—567) | 977 (898—1056) | 416 (409—423) | 840 (767—914) | 486 (480—492) | 908 (854—962) |
| **2012** | 521 (512—529) | 918 (852—984) | 372 (365—379) | 776 (711—842) | 445 (440—450) | 846 (800—893) |
| **2013** | 501 (493—509) | 973 (907—1039) | 363 (356—370) | 772 (708—836) | 431 (426—436) | 871 (825—917) |
| **2014** | 504 (496—512) | 1001 (937—1065) | 356 (349—362) | 827 (762—892) | 429 (423—434) | 913 (867—958) |
| **2015** | 550 (542—559) | 1064 (1000—1128) | 388 (381—395) | 826 (767—885) | 468 (463—473) | 943 (900—987) |
| **2016** | 577 (568—585) | 1065 (1008—1123) | 409 (402—416) | 865 (808—923) | 492 (486—497) | 964 (923—1004) |
| **2017** | 584 (576—593) | 1110 (1051—1168) | 415 (408—422) | 926 (869—983) | 498 (493—504) | 1016 (976—1057) |
| **2018** | 564 (556—573) | 1012 (960—1064) | 409 (402—416) | 868 (814—922) | 486 (480—491) | 939 (901—976) |
| **2019** | 570 (562—578) | 1035 (985—1085) | 410 (403—417) | 850 (799—900) | 489 (483—494) | 941 (905—976) |
| **2020** | 524 (516—532) | 1067 (1017—1116) | 381 (375—388) | 800 (752—848) | 452 (447—457) | 931 (897—966) |
| **2021** | 605 (596—613) | 1143 (1092—1193) | 457 (450—464) | 936 (886—985) | 530 (524—536) | 1037 (1002—1073) |
| **Trend %** | 0.1 (-0.5—0.8) | 0.8 (0.3—1.4) | -0.4 (-1.3—0.5) | 0.1 (-0.7—0.9) | -0.1 (-0.8—0.6) | 0.5 (-0.2—1.2) |
| **2021** | 7.97 (7.94—8.00) | 14.76 (14.61—14.92) | 6.02 (5.99—6.04) | 13.17 (13.00—13.33) | 6.99 (6.97—7.01) | 13.96 (13.85—14.07) |

24

For Review Only

Figure S1. Incidence (per 100 000) of type 2 diabetes 2006 and 2021 by age in men and women.

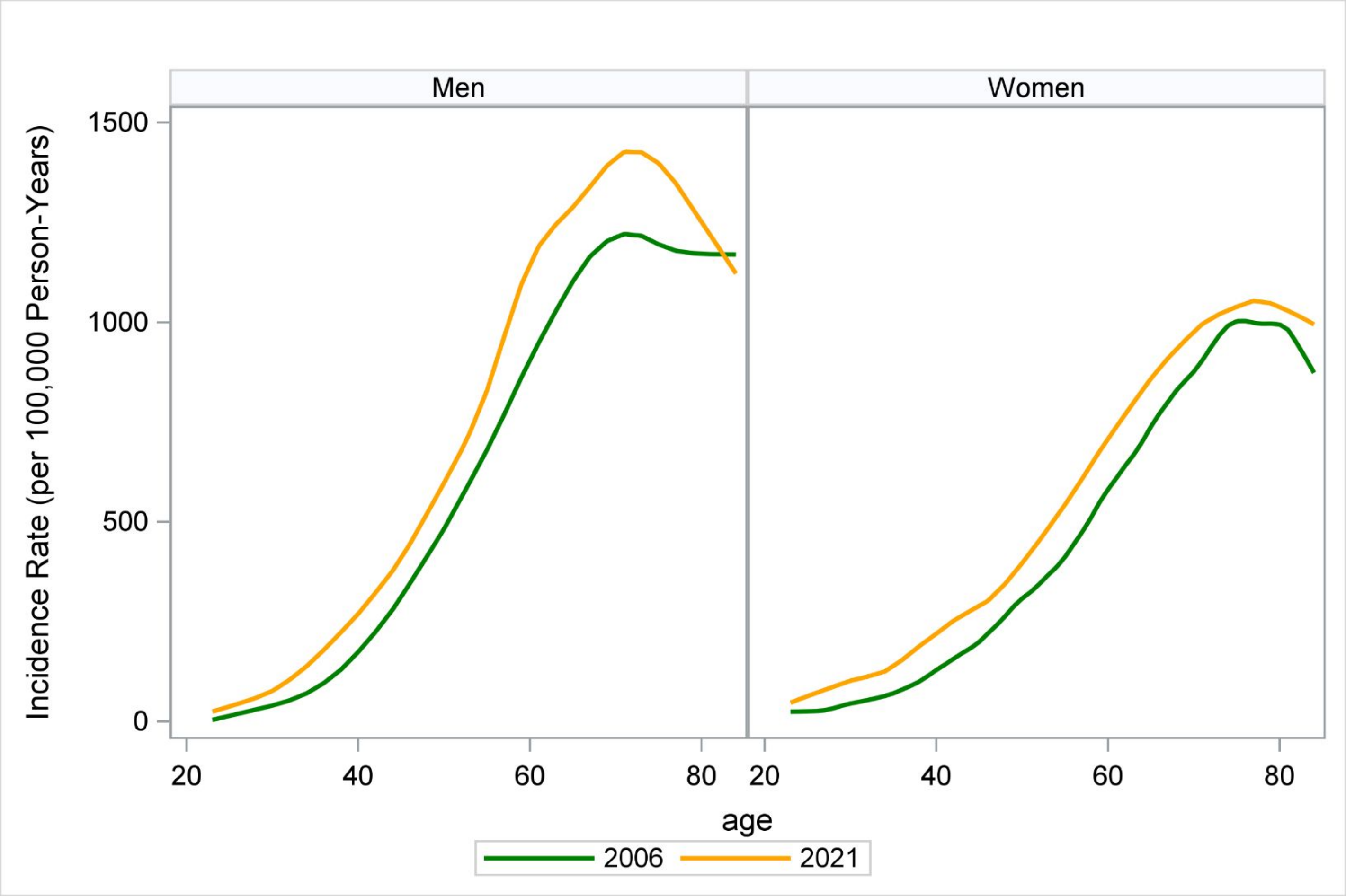
Men
Women
Incidence Rate (per 100,000 Person-Years)
1500
1000
500
0
20
40
60
80
age
2006
2021

Figure S2. Age-standardized incidence (per 100 000) of type 2 diabetes 2006 to 2021by birth region.

*Nordic countries except Sweden, **Europe except the Nordic countries.

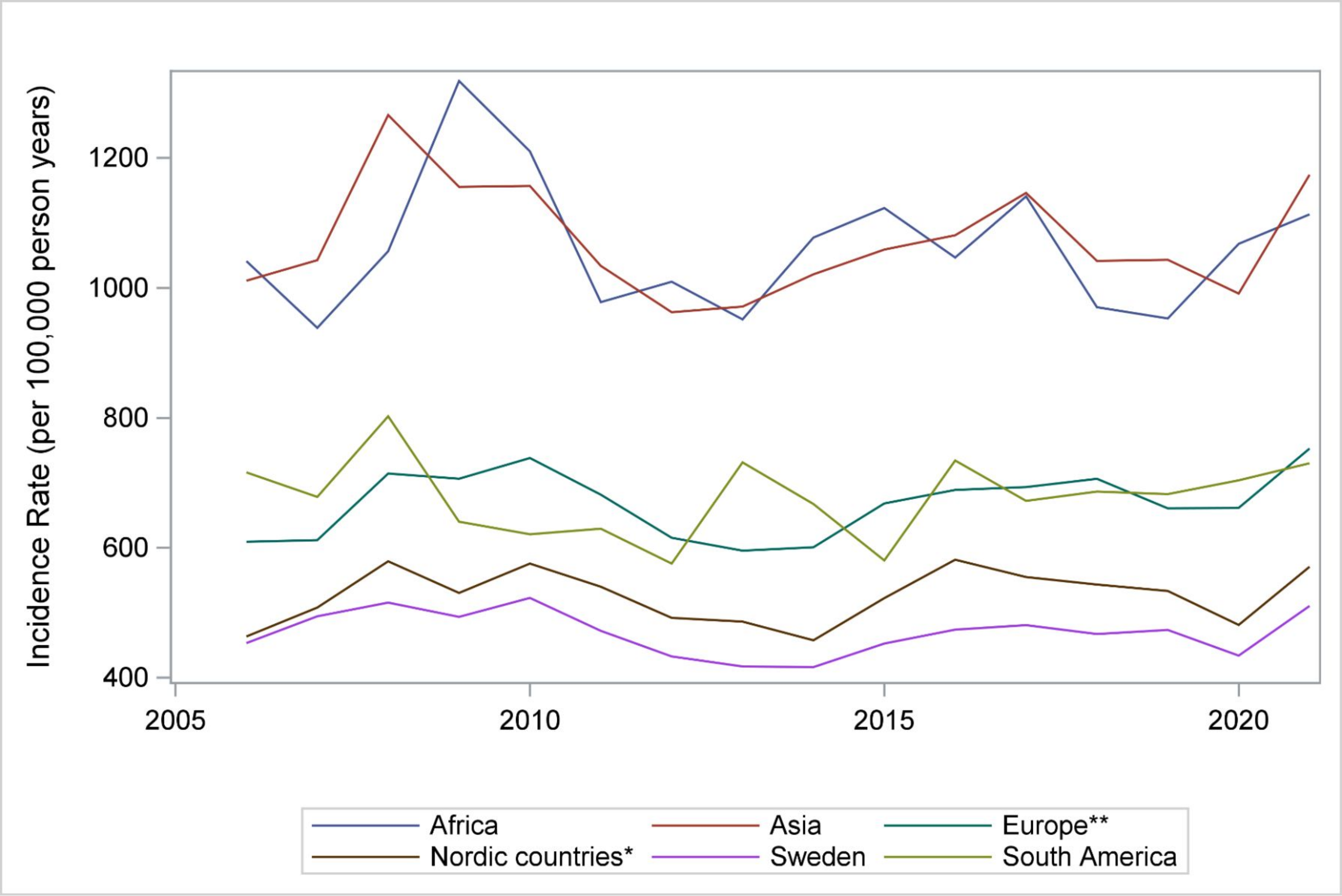

**Table S6. Age-standardized prevalence (%) with 95% confidence intervals of type 2 diabetes 2006 to 2021, by educational level.**

| | Men | | | Women | | | Total | | |
|---|---|---|---|---|---|---|---|---|---|
| **Year** | Primary | Secondary | Tertiary | Primary | Secondary | Tertiary | Primary | Secondary | Tertiary |
| **2006** | 6.13 (6.08—6.18) | 5.36 (5.32—5.41) | 4.09 (4.03—4.14) | 5.38 (5.33—5.42) | 4.06 (4.02—4.10) | 2.58 (2.53—2.63) | 5.75 (5.72—5.78) | 4.71 (4.68—4.74) | 3.33 (3.29—3.37) |
| **2007** | 6.55 (6.50—6.60) | 5.71 (5.67—5.75) | 4.39 (4.33—4.44) | 5.77 (5.72—5.82) | 4.36 (4.32—4.40) | 2.78 (2.73—2.83) | 6.16 (6.12—6.19) | 5.03 (5.00—5.06) | 3.58 (3.54—3.62) |
| **2008** | 6.99 (6.94—7.04) | 6.08 (6.04—6.13) | 4.68 (4.62—4.73) | 6.19 (6.14—6.24) | 4.68 (4.64—4.72) | 2.98 (2.93—3.03) | 6.59 (6.55—6.62) | 5.38 (5.35—5.41) | 3.83 (3.79—3.86) |
| **2009** | 7.37 (7.32—7.42) | 6.41 (6.36—6.45) | 4.92 (4.86—4.97) | 6.56 (6.50—6.61) | 4.95 (4.91—4.99) | 3.15 (3.10—3.20) | 6.96 (6.93—7.00) | 5.68 (5.65—5.70) | 4.03 (3.99—4.07) |
| **2010** | 7.77 (7.72—7.82) | 6.73 (6.68—6.77) | 5.15 (5.10—5.20) | 6.93 (6.87—6.98) | 5.20 (5.16—5.24) | 3.32 (3.27—3.36) | 7.35 (7.31—7.39) | 5.96 (5.93—5.99) | 4.23 (4.19—4.27) |
| **2011** | 8.07 (8.01—8.12) | 6.96 (6.92—7.01) | 5.34 (5.29—5.39) | 7.22 (7.16—7.28) | 5.39 (5.35—5.43) | 3.44 (3.40—3.49) | 7.64 (7.60—7.68) | 6.17 (6.14—6.20) | 4.39 (4.35—4.42) |
| **2012** | 8.34 (8.28—8.39) | 7.16 (7.12—7.21) | 5.47 (5.42—5.52) | 7.45 (7.39—7.51) | 5.53 (5.49—5.57) | 3.53 (3.49—3.58) | 7.89 (7.85—7.93) | 6.34 (6.31—6.37) | 4.50 (4.46—4.53) |
| **2013** | 8.57 (8.51—8.63) | 7.33 (7.29—7.37) | 5.58 (5.52—5.63) | 7.68 (7.62—7.74) | 5.65 (5.61—5.68) | 3.61 (3.57—3.66) | 8.12 (8.08—8.16) | 6.49 (6.46—6.51) | 4.59 (4.56—4.62) |
| **2014** | 8.82 (8.76—8.88) | 7.50 (7.46—7.54) | 5.66 (5.61—5.71) | 7.89 (7.83—7.95) | 5.75 (5.71—5.79) | 3.69 (3.65—3.74) | 8.35 (8.31—8.40) | 6.62 (6.59—6.65) | 4.67 (4.64—4.71) |
| **2015** | 9.11 (9.05—9.17) | 7.70 (7.66—7.74) | 5.80 (5.75—5.85) | 8.14 (8.08—8.21) | 5.90 (5.86—5.94) | 3.78 (3.74—3.83) | 8.63 (8.58—8.67) | 6.80 (6.77—6.83) | 4.79 (4.75—4.82) |
| **2016** | 9.45 (9.38—9.51) | 7.91 (7.87—7.95) | 5.95 (5.90—6.00) | 8.45 (8.38—8.51) | 6.05 (6.02—6.09) | 3.89 (3.85—3.93) | 8.95 (8.90—8.99) | 6.98 (6.95—7.01) | 4.92 (4.89—4.95) |
| **2017** | 9.77 (9.71—9.84) | 8.13 (8.09—8.17) | 6.09 (6.04—6.14) | 8.77 (8.71—8.84) | 6.23 (6.19—6.27) | 4.00 (3.96—4.04) | 9.27 (9.22—9.32) | 7.17 (7.15—7.20) | 5.04 (5.01—5.07) |
| **2018** | 10.06 (10.00—10.13) | 8.30 (8.25—8.34) | 6.22 (6.18—6.27) | 9.06 (8.99—9.14) | 6.38 (6.34—6.42) | 4.11 (4.07—4.15) | 9.56 (9.51—9.61) | 7.33 (7.30—7.36) | 5.16 (5.13—5.19) |
| **2019** | 10.34 (10.27—10.40) | 8.49 (8.45—8.53) | 6.33 (6.28—6.38) | 9.33 (9.26—9.41) | 6.51 (6.48—6.55) | 4.23 (4.19—4.26) | 9.83 (9.78—9.88) | 7.50 (7.47—7.53) | 5.27 (5.24—5.30) |
| **2020** | 10.58 (10.51—10.65) | 8.61 (8.57—8.65) | 6.41 (6.37—6.46) | 9.58 (9.50—9.65) | 6.64 (6.60—6.68) | 4.30 (4.26—4.34) | 10.08 (10.03—10.13) | 7.62 (7.59—7.65) | 5.35 (5.32—5.38) |

| | | | | | | | | | |
|---|---|---|---|---|---|---|---|---|---|
| **2021** | 10.93 (10.86—11.00) | 8.84 (8.80—8.88) | 6.57 (6.52—6.61) | 9.93 (9.85—10.01) | 6.84 (6.80—6.88) | 4.46 (4.42—4.49) | 10.43 (10.37—10.48) | 7.83 (7.81—7.86) | 5.51 (5.48—5.54) |

**Table S7. Age-standardized incidence (per 100 000) with 95% confidence intervals of type 2 diabetes 2006 to 2021, by educational level.**

| | Men | | | Women | | | Total | | |
|---|---|---|---|---|---|---|---|---|---|
| **Year** | Primary | Secondary | Tertiary | Primary | Secondary | Tertiary | Primary | Secondary | Tertiary |
| **2006** | 635 (618—653) | 537 (523—551) | 385 (368—401) | 544 (526—562) | 421 (409—434) | 277 (261—293) | 589 (576—601) | 478 (469—488) | 330 (319—341) |
| **2007** | 685 (667—703) | 578 (564—593) | 455 (438—473) | 596 (578—615) | 454 (442—467) | 305 (289—320) | 640 (627—653) | 515 (506—525) | 379 (367—390) |
| **2008** | 741 (721—760) | 632 (617—647) | 478 (461—495) | 652 (633—672) | 493 (480—506) | 311 (296—326) | 696 (682—709) | 561 (552—571) | 393 (382—405) |
| **2009** | 714 (695—733) | 619 (604—633) | 460 (444—477) | 637 (617—657) | 468 (455—480) | 298 (284—312) | 675 (661—688) | 542 (533—551) | 378 (367—389) |
| **2010** | 794 (773—814) | 656 (641—670) | 480 (464—496) | 688 (667—710) | 490 (478—503) | 325 (311—339) | 740 (725—755) | 572 (562—581) | 401 (391—412) |
| **2011** | 705 (686—725) | 596 (583—610) | 462 (446—477) | 630 (609—651) | 453 (441—465) | 293 (280—306) | 667 (652—681) | 524 (515—533) | 376 (366—386) |
| **2012** | 696 (676—716) | 564 (551—577) | 411 (397—426) | 567 (547—587) | 414 (403—425) | 268 (256—280) | 630 (616—644) | 488 (479—496) | 339 (329—348) |
| **2013** | 681 (661—701) | 548 (535—560) | 398 (384—412) | 578 (558—599) | 396 (385—407) | 265 (253—276) | 629 (615—643) | 471 (462—479) | 330 (321—339) |
| **2014** | 694 (674—715) | 559 (547—572) | 395 (382—408) | 578 (557—599) | 391 (380—401) | 269 (258—281) | 635 (621—650) | 474 (466—482) | 331 (323—340) |
| **2015** | 754 (733—776) | 612 (599—625) | 440 (426—454) | 640 (618—662) | 444 (433—455) | 281 (270—292) | 696 (681—712) | 527 (518—535) | 359 (350—368) |
| **2016** | 820 (797—842) | 639 (625—652) | 465 (451—479) | 693 (670—717) | 452 (441—463) | 308 (297—319) | 756 (739—772) | 544 (535—552) | 385 (376—394) |
| **2017** | 843 (819—866) | 650 (637—663) | 462 (448—475) | 715 (691—739) | 480 (469—492) | 314 (303—325) | 778 (761—795) | 564 (555—573) | 387 (378—395) |
| **2018** | 821 (798—845) | 619 (607—632) | 474 (460—487) | 692 (668—716) | 468 (457—479) | 324 (313—335) | 756 (739—773) | 542 (534—551) | 398 (389—406) |
| **2019** | 809 (785—832) | 647 (634—660) | 466 (453—479) | 705 (680—730) | 471 (460—482) | 331 (320—342) | 756 (739—773) | 558 (549—566) | 397 (389—406) |
| **2020** | 785 (761—809) | 595 (583—607) | 440 (428—453) | 680 (655—705) | 452 (441—463) | 295 (285—304) | 732 (715—749) | 522 (514—530) | 366 (358—374) |
| **2021** | 900 | 686 | 499 | 785 | 534 | 371 | 842 | 609 | 434 |

| | (874-925) | (673-699) | (486-513) | (759-812) | (522-546) | (360-382) | (823-860) | (600-617) | (426-443) |
|---|---|---|---|---|---|---|---|---|---|
| **Annual change % (95% CI)** | 1.6 (0.9—2.2) | 0.7 (0.1—1.4) | 0.6 (-0.2—1.4) | 1.4 (0.7—2.2) | 0.5 (-0.3—1.4) | 1.1 (0.2—2.1) | 1.5 (0.8—2.2) | 0.7 (-0.1—1.4) | 0.8 (-0.0—1.6) |

For Review Only

| Table S8. Age-standardized prevalence (%) of early-onset type 2 diabetes 2006 to 2021, by birth region (Europe vs. outside Europe) | | | | | | |
|---|---|---|---|---|---|---|
| | Men | | Women | | Total | |
| **Year** | Born in Europe | Born outside Europe | Born in Europe | Born outside Europe | Born in Europe | Born outside Europe |
| **2006** | 0.22 (0.21—0.23) | 0.62 (0.57—0.68) | 0.24 (0.23—0.25) | 0.72 (0.66—0.77) | 0.23 (0.22—0.24) | 0.67 (0.63—0.71) |
| **2007** | 0.23 (0.22—0.24) | 0.65 (0.59—0.70) | 0.25 (0.24—0.26) | 0.75 (0.69—0.80) | 0.24 (0.23—0.25) | 0.70 (0.66—0.73) |
| **2008** | 0.24 (0.23—0.25) | 0.71 (0.65—0.76) | 0.27 (0.26—0.28) | 0.78 (0.73—0.84) | 0.25 (0.25—0.26) | 0.75 (0.71—0.78) |
| **2009** | 0.25 (0.24—0.26) | 0.73 (0.68—0.79) | 0.28 (0.26—0.29) | 0.83 (0.78—0.88) | 0.26 (0.26—0.27) | 0.78 (0.74—0.82) |
| **2010** | 0.27 (0.26—0.29) | 0.78 (0.73—0.83) | 0.29 (0.28—0.30) | 0.91 (0.86—0.96) | 0.28 (0.28—0.29) | 0.84 (0.81—0.88) |
| **2011** | 0.29 (0.28—0.30) | 0.82 (0.76—0.87) | 0.31 (0.30—0.32) | 0.93 (0.87—0.98) | 0.30 (0.29—0.31) | 0.87 (0.83—0.91) |
| **2012** | 0.30 (0.29—0.31) | 0.83 (0.77—0.88) | 0.31 (0.30—0.33) | 0.95 (0.89—1.00) | 0.31 (0.30—0.32) | 0.88 (0.85—0.92) |
| **2013** | 0.31 (0.30—0.32) | 0.85 (0.80—0.90) | 0.32 (0.31—0.33) | 0.96 (0.91—1.01) | 0.31 (0.31—0.32) | 0.90 (0.87—0.94) |
| **2014** | 0.32 (0.31—0.33) | 0.86 (0.82—0.91) | 0.32 (0.31—0.34) | 0.96 (0.92—1.01) | 0.32 (0.31—0.33) | 0.91 (0.88—0.95) |
| **2015** | 0.33 (0.32—0.34) | 0.87 (0.83—0.92) | 0.33 (0.32—0.35) | 0.97 (0.93—1.02) | 0.33 (0.32—0.34) | 0.92 (0.89—0.95) |
| **2016** | 0.35 (0.34—0.36) | 0.89 (0.85—0.93) | 0.35 (0.34—0.36) | 1.00 (0.96—1.05) | 0.35 (0.34—0.36) | 0.94 (0.91—0.98) |
| **2017** | 0.37 (0.36—0.38) | 0.93 (0.88—0.97) | 0.37 (0.36—0.39) | 1.08 (1.03—1.12) | 0.37 (0.36—0.38) | 1.00 (0.97—1.03) |
| **2018** | 0.39 (0.38—0.40) | 0.98 (0.93—1.02) | 0.41 (0.40—0.42) | 1.20 (1.15—1.26) | 0.40 (0.39—0.41) | 1.09 (1.05—1.12) |
| **2019** | 0.42 (0.40—0.43) | 1.02 (0.98—1.07) | 0.46 (0.45—0.47) | 1.29 (1.24—1.34) | 0.44 (0.43—0.45) | 1.15 (1.12—1.19) |
| **2020** | 0.45 (0.43—0.46) | 1.02 (0.98—1.07) | 0.50 (0.48—0.51) | 1.36 (1.31—1.42) | 0.47 (0.46—0.48) | 1.19 (1.15—1.22) |
| **2021** | 0.48 (0.47—0.50) | 1.08 (1.03—1.13) | 0.55 (0.54—0.57) | 1.46 (1.40—1.52) | 0.52 (0.51—0.53) | 1.26 (1.23—1.30) |

**Table S9. Age-standardized incidence (per 100 000) with 95% confidence intervals of early-onset type 2 diabetes 2006 to 2021, by birth region (Europe vs. outside Europe)**

| | Men | | Women | | Total | |
|---|---|---|---|---|---|---|
| **Year** | Born in Europe | Born outside Europe | Born in Europe | Born outside Europe | Born in Europe | Born outside Europe |
| **2006** | 46 (42—50) | 158 (129—186) | 46 (41—50) | 137 (113—162) | 46 (43—49) | 148 (129—166) |
| **2007** | 46 (42—51) | 150 (124—177) | 42 (38—47) | 141 (117—164) | 44 (41—47) | 145 (128—163) |
| **2008** | 53 (48—57) | 184 (156—213) | 42 (38—47) | 141 (118—164) | 48 (45—51) | 163 (145—182) |
| **2009** | 52 (47—56) | 175 (149—201) | 40 (36—44) | 157 (134—181) | 46 (43—49) | 166 (149—184) |
| **2010** | 61 (56—66) | 206 (179—234) | 48 (44—53) | 187 (162—211) | 55 (51—58) | 197 (178—215) |
| **2011** | 60 (55—65) | 173 (149—197) | 53 (48—58) | 151 (129—172) | 57 (53—60) | 162 (146—179) |
| **2012** | 54 (49—59) | 152 (130—174) | 38 (34—42) | 125 (106—144) | 46 (43—49) | 139 (124—154) |
| **2013** | 52 (48—57) | 170 (148—192) | 36 (32—40) | 148 (128—168) | 44 (41—48) | 159 (144—174) |
| **2014** | 55 (50—59) | 160 (139—180) | 40 (36—44) | 143 (124—162) | 47 (44—51) | 152 (138—166) |
| **2015** | 57 (52—62) | 180 (159—201) | 43 (38—47) | 148 (130—167) | 50 (47—53) | 165 (151—179) |
| **2016** | 64 (59—69) | 183 (164—203) | 49 (45—54) | 182 (162—201) | 57 (53—60) | 182 (169—196) |
| **2017** | 67 (62—73) | 190 (170—209) | 53 (48—58) | 178 (158—198) | 60 (57—64) | 184 (170—198) |
| **2018** | 65 (60—70) | 193 (173—213) | 69 (64—75) | 220 (198—242) | 67 (63—71) | 206 (191—221) |
| **2019** | 70 (64—75) | 207 (186—227) | 81 (75—87) | 204 (182—226) | 75 (71—79) | 205 (190—220) |
| **2020** | 75 (69—80) | 178 (159—197) | 76 (71—82) | 203 (181—226) | 76 (72—80) | 190 (176—205) |
| **2021** | 87 (81—93) | 204 (183—225) | 91 (85—97) | 218 (195—241) | 89 (84—93) | 211 (195—226) |
| **Annual change % (95% CI)** | 3.4 (2.6—4.3) | 1.4 (0.5—2.2) | 5.4 (3.0—7.9) | 3.4 (2.0—4.8) | 4.3 (2.9—5.7) | 2.3 (1.3—3.4) |

Figure S3. Incidence (per 100 000) with 95% confidence intervals of early-onset type 2 diabetes 2006 to 2021 by sex in people born within Europe.

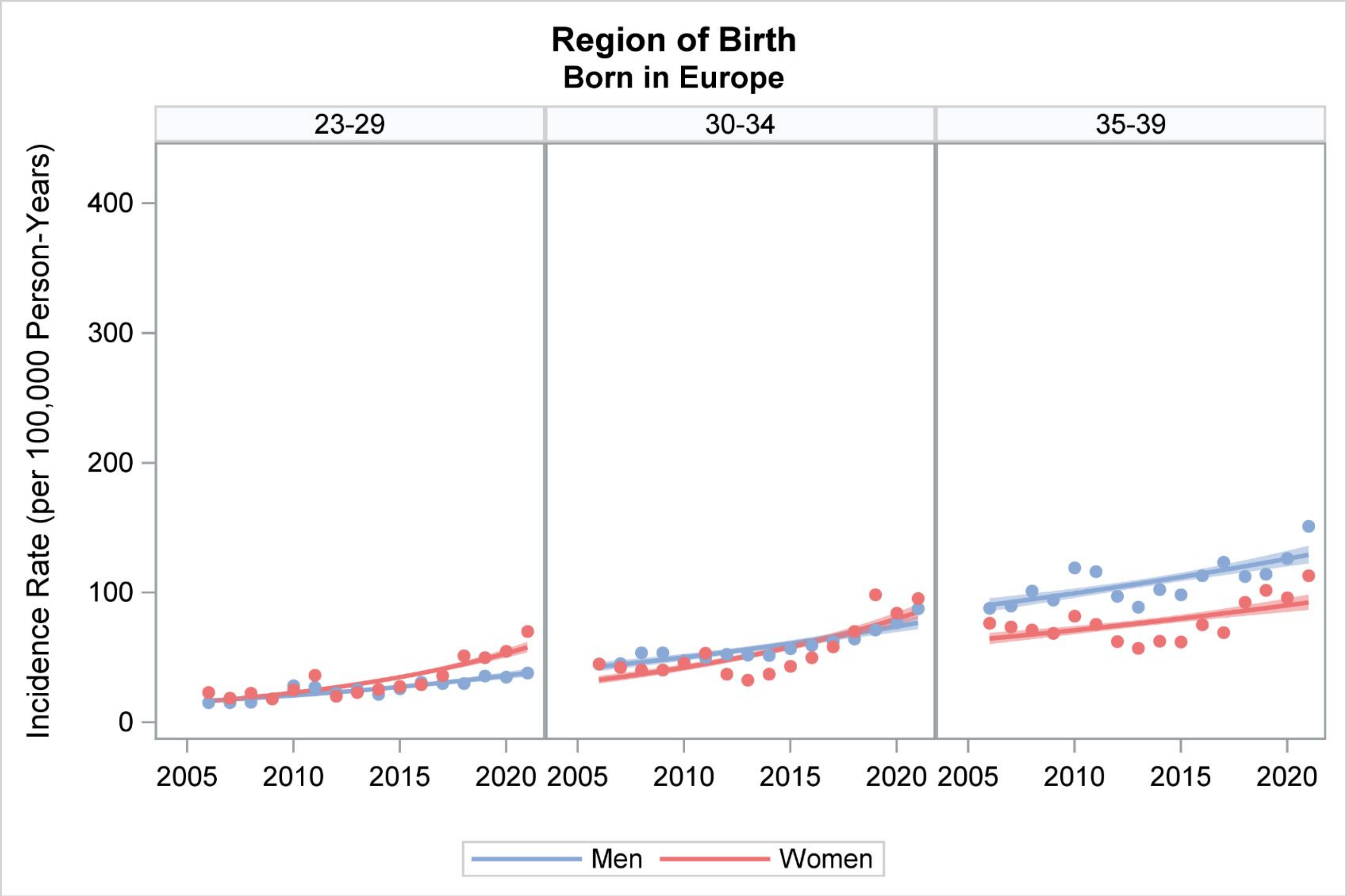

Figure S4. Incidence (per 100 000) with 95% confidence intervals of early-onset type 2 diabetes 2006 to 2021 by sex in people born outside of Europe.

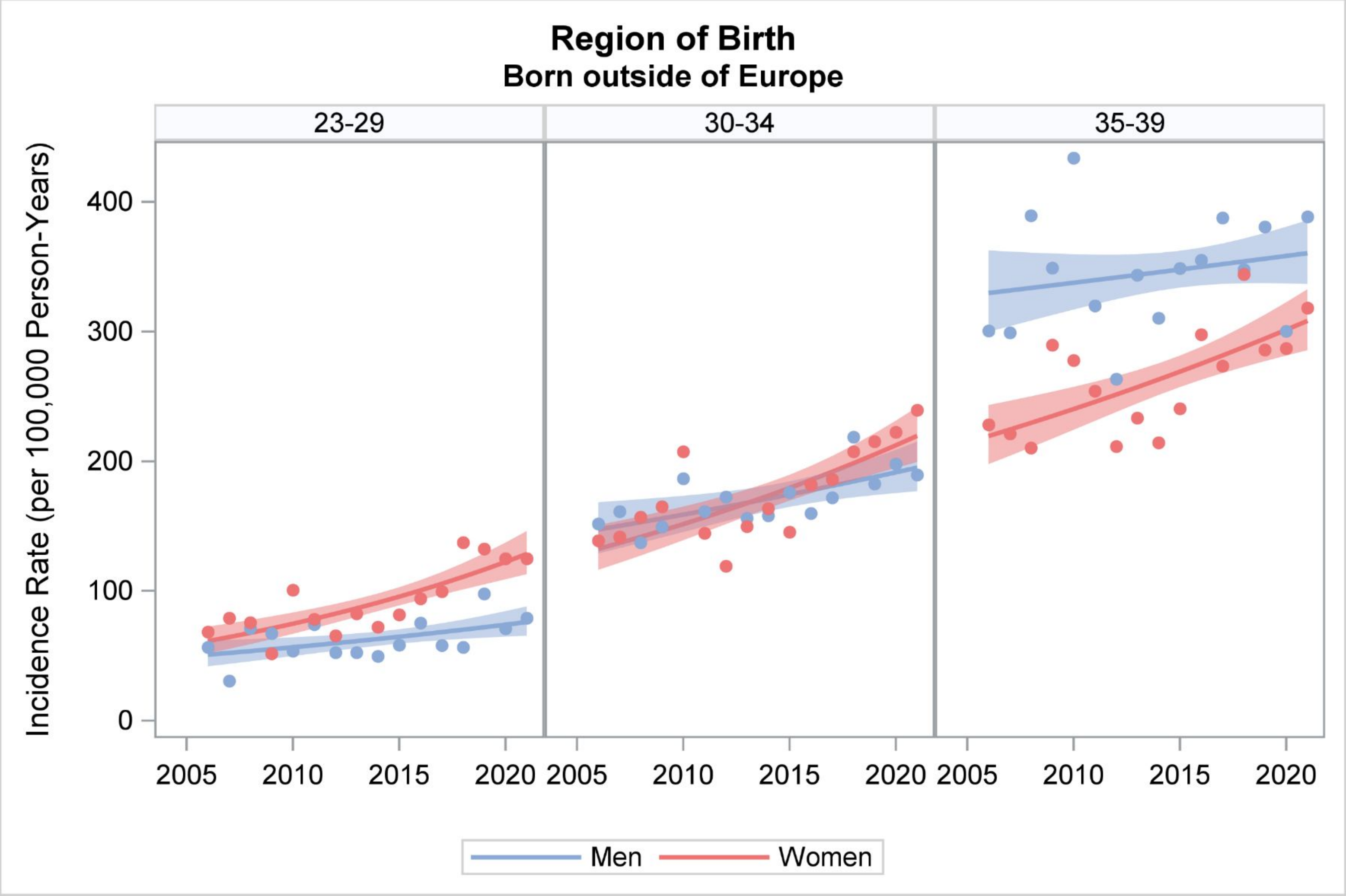

**Table S10. Age-standardized prevalence (%) with 95% confidence intervals of early-onset type 2 diabetes 2006 to 2021 by educational level.**

| | Men | | | Women | | | Total | | |
|---|---|---|---|---|---|---|---|---|---|
| **Year** | Primary | Secondary | Tertiary | Primary | Secondary | Tertiary | Primary | Secondary | Tertiary |
| **2006** | 0.51 (0.46—0.55) | 0.29 (0.27—0.30) | 0.13 (0.12—0.14) | 0.76 (0.70—0.82) | 0.36 (0.34—0.38) | 0.16 (0.14—0.17) | 0.63 (0.59—0.67) | 0.32 (0.31—0.34) | 0.14 (0.14—0.15) |
| **2007** | 0.52 (0.47—0.56) | 0.30 (0.29—0.32) | 0.14 (0.13—0.15) | 0.80 (0.73—0.86) | 0.39 (0.37—0.41) | 0.16 (0.15—0.17) | 0.65 (0.61—0.69) | 0.35 (0.33—0.36) | 0.15 (0.15—0.16) |
| **2008** | 0.57 (0.52—0.61) | 0.33 (0.31—0.34) | 0.16 (0.14—0.17) | 0.86 (0.80—0.93) | 0.42 (0.40—0.44) | 0.17 (0.16—0.18) | 0.71 (0.67—0.75) | 0.37 (0.36—0.39) | 0.16 (0.15—0.17) |
| **2009** | 0.60 (0.55—0.65) | 0.34 (0.33—0.36) | 0.17 (0.15—0.18) | 0.93 (0.86—0.99) | 0.44 (0.42—0.46) | 0.18 (0.17—0.19) | 0.76 (0.72—0.80) | 0.39 (0.38—0.40) | 0.17 (0.16—0.18) |
| **2010** | 0.66 (0.61—0.71) | 0.38 (0.36—0.39) | 0.18 (0.17—0.20) | 1.03 (0.96—1.10) | 0.48 (0.46—0.51) | 0.19 (0.18—0.20) | 0.84 (0.80—0.88) | 0.43 (0.41—0.44) | 0.19 (0.18—0.20) |
| **2011** | 0.71 (0.66—0.76) | 0.40 (0.39—0.42) | 0.20 (0.19—0.21) | 1.11 (1.03—1.18) | 0.51 (0.48—0.53) | 0.21 (0.19—0.22) | 0.90 (0.86—0.95) | 0.45 (0.44—0.47) | 0.20 (0.19—0.21) |
| **2012** | 0.76 (0.71—0.82) | 0.42 (0.40—0.44) | 0.20 (0.19—0.21) | 1.09 (1.02—1.17) | 0.52 (0.50—0.54) | 0.22 (0.20—0.23) | 0.92 (0.88—0.97) | 0.47 (0.45—0.48) | 0.21 (0.20—0.22) |
| **2013** | 0.76 (0.71—0.81) | 0.43 (0.41—0.45) | 0.22 (0.21—0.23) | 1.12 (1.05—1.20) | 0.53 (0.51—0.56) | 0.22 (0.21—0.24) | 0.94 (0.89—0.98) | 0.48 (0.46—0.50) | 0.22 (0.21—0.23) |
| **2014** | 0.78 (0.73—0.83) | 0.45 (0.43—0.46) | 0.23 (0.22—0.25) | 1.14 (1.07—1.21) | 0.53 (0.51—0.56) | 0.23 (0.22—0.25) | 0.96 (0.91—1.00) | 0.49 (0.47—0.50) | 0.23 (0.23—0.24) |

For Review Only

| | | | | | | | | | |
|---|---|---|---|---|---|---|---|---|---|
| **2015** | 0.81 (0.76—0.86) | 0.46 (0.44—0.48) | 0.25 (0.23—0.26) | 1.19 (1.12—1.26) | 0.55 (0.53—0.58) | 0.24 (0.23—0.26) | 0.99 (0.95—1.04) | 0.50 (0.49—0.52) | 0.25 (0.24—0.26) |
| **2016** | 0.86 (0.81—0.91) | 0.48 (0.46—0.50) | 0.27 (0.25—0.28) | 1.23 (1.16—1.30) | 0.58 (0.55—0.60) | 0.26 (0.25—0.28) | 1.04 (1.00—1.08) | 0.53 (0.51—0.54) | 0.26 (0.25—0.27) |
| **2017** | 0.93 (0.88—0.98) | 0.50 (0.48—0.52) | 0.28 (0.27—0.30) | 1.30 (1.22—1.37) | 0.61 (0.58—0.63) | 0.28 (0.27—0.30) | 1.11 (1.07—1.15) | 0.55 (0.54—0.57) | 0.28 (0.27—0.29) |
| **2018** | 1.01 (0.96—1.07) | 0.52 (0.50—0.54) | 0.30 (0.28—0.31) | 1.42 (1.34—1.50) | 0.65 (0.62—0.68) | 0.32 (0.30—0.33) | 1.21 (1.16—1.26) | 0.58 (0.57—0.60) | 0.31 (0.30—0.32) |
| **2019** | 1.07 (1.01—1.12) | 0.55 (0.53—0.57) | 0.31 (0.30—0.33) | 1.50 (1.42—1.58) | 0.70 (0.67—0.73) | 0.37 (0.35—0.38) | 1.27 (1.23—1.32) | 0.62 (0.60—0.64) | 0.34 (0.33—0.35) |
| **2020** | 1.11 (1.05—1.16) | 0.55 (0.53—0.57) | 0.33 (0.31—0.35) | 1.52 (1.44—1.60) | 0.74 (0.72—0.77) | 0.39 (0.38—0.41) | 1.31 (1.26—1.36) | 0.64 (0.63—0.66) | 0.36 (0.35—0.37) |
| **2021** | 1.12 (1.07—1.18) | 0.59 (0.57—0.61) | 0.36 (0.34—0.38) | 1.50 (1.42—1.58) | 0.80 (0.78—0.83) | 0.43 (0.41—0.45) | 1.31 (1.26—1.35) | 0.69 (0.67—0.71) | 0.40 (0.38—0.41) |

| Table S11. Age-standardized incidence (per 100 000) with 95% confidence intervals of early-onset type 2 diabetes 2006 to 2021 by educational level. | | | | | | | | | |
|---|---|---|---|---|---|---|---|---|---|
| | Men | | | Women | | | Total | | |
| **Year** | Primary | Secondary | Tertiary | Primary | Secondary | Tertiary | Primary | Secondary | Tertiary |
| **2006** | 120 (099—142) | 62 (55—69) | 29 (24—34) | 150 (121—178) | 63 (55—71) | 33 (28—38) | 135 (117—152) | 62 (57—68) | 31 (27—35) |
| **2007** | 106 (086—126) | 60 (54—67) | 34 (28—40) | 128 (101—155) | 70 (62—79) | 28 (23—33) | 117 (100—133) | 65 (60—71) | 31 (27—35) |
| **2008** | 142 (119—165) | 71 (64—79) | 36 (30—41) | 146 (118—174) | 66 (58—75) | 30 (25—35) | 144 (126—162) | 69 (63—75) | 33 (29—37) |
| **2009** | 132 (110—154) | 72 (64—80) | 35 (29—40) | 160 (131—189) | 65 (57—73) | 29 (25—34) | 146 (128—164) | 69 (63—74) | 32 (29—36) |
| **2010** | 158 (134—182) | 84 (76—92) | 45 (39—51) | 196 (164—228) | 81 (72—91) | 35 (30—40) | 176 (157—196) | 83 (76—89) | 40 (36—44) |
| **2011** | 146 (123—169) | 82 (74—90) | 44 (38—50) | 196 (165—228) | 82 (73—92) | 37 (32—42) | 171 (151—190) | 82 (76—88) | 40 (36—44) |
| **2012** | 148 (125—171) | 74 (66—81) | 34 (29—40) | 121 (096—145) | 64 (56—73) | 28 (24—33) | 135 (118—152) | 69 (63—75) | 31 (28—35) |
| **2013** | 137 (115—159) | 73 (65—81) | 43 (37—50) | 167 (138—196) | 61 (52—69) | 29 (25—33) | 152 (134—169) | 67 (61—73) | 36 (33—40) |
| **2014** | 149 (127—171) | 74 (66—82) | 41 (35—47) | 159 (132—187) | 67 (58—76) | 31 (26—35) | 154 (136—171) | 71 (65—76) | 36 (32—40) |
| **2015** | 146 (124—167) | 86 (78—95) | 45 (39—52) | 185 (157—214) | 71 (62—81) | 33 (28—38) | 165 (147—183) | 79 (73—85) | 39 (35—43) |
| **2016** | 171 (149—194) | 87 (79—96) | 52 (45—59) | 196 (167—226) | 82 (73—92) | 44 (38—49) | 183 (165—202) | 85 (79—91) | 48 (44—52) |
| **2017** | 181 (158—204) | 93 (84—1.02) | 55 (48—61) | 180 (153—208) | 86 (77—96) | 45 (40—51) | 181 (163—198) | 90 (83—96) | 50 (46—54) |
| **2018** | 183 (159—206) | 85 (77—94) | 58 (50—65) | 215 (185—246) | 107 (96—118) | 61 (54—67) | 199 (180—218) | 96 (89—1.03) | 59 (54—64) |
| **2019** | 166 (144—188) | 97 (89—1.06) | 62 (54—69) | 208 (178—238) | 111 (100—122) | 71 (64—78) | 186 (168—205) | 104 (97—111) | 66 (61—71) |
| **2020** | 186 (163—209) | 93 (85—1.02) | 58 (50—65) | 197 (168—226) | 117 (106—128) | 59 (52—65) | 191 (173—209) | 105 (98—112) | 58 (53—63) |
| **2021** | 204 (180—227) | 1.07 (98—1.16) | 72 (63—81) | 204 (176—233) | 125 (114—136) | 69 (62—77) | 204 (185—223) | 115 (108—122) | 71 (65—77) |

| | | | | | | | | | |
|---|---|---|---|---|---|---|---|---|---|
| **Annual change % (95% CI)** | 3.3 (2.5—4.1) | 3.1 (2.3—3.8) | 5.2 (4.2—6.3) | 2.7 (1.4—4.0) | 4.7 (3.1—6.3) | 6.9 (4.5—9.3) | 3.0 (2.1—3.8) | 3.8 (2.9—4.8) | 5.9 (4.5—7.4) |

For Review Only

Figure S5. Incidence (per 100 000) with 95% confidence intervals of early-onset type 2 diabetes 2006 to 2021 by sex in people with primary education.

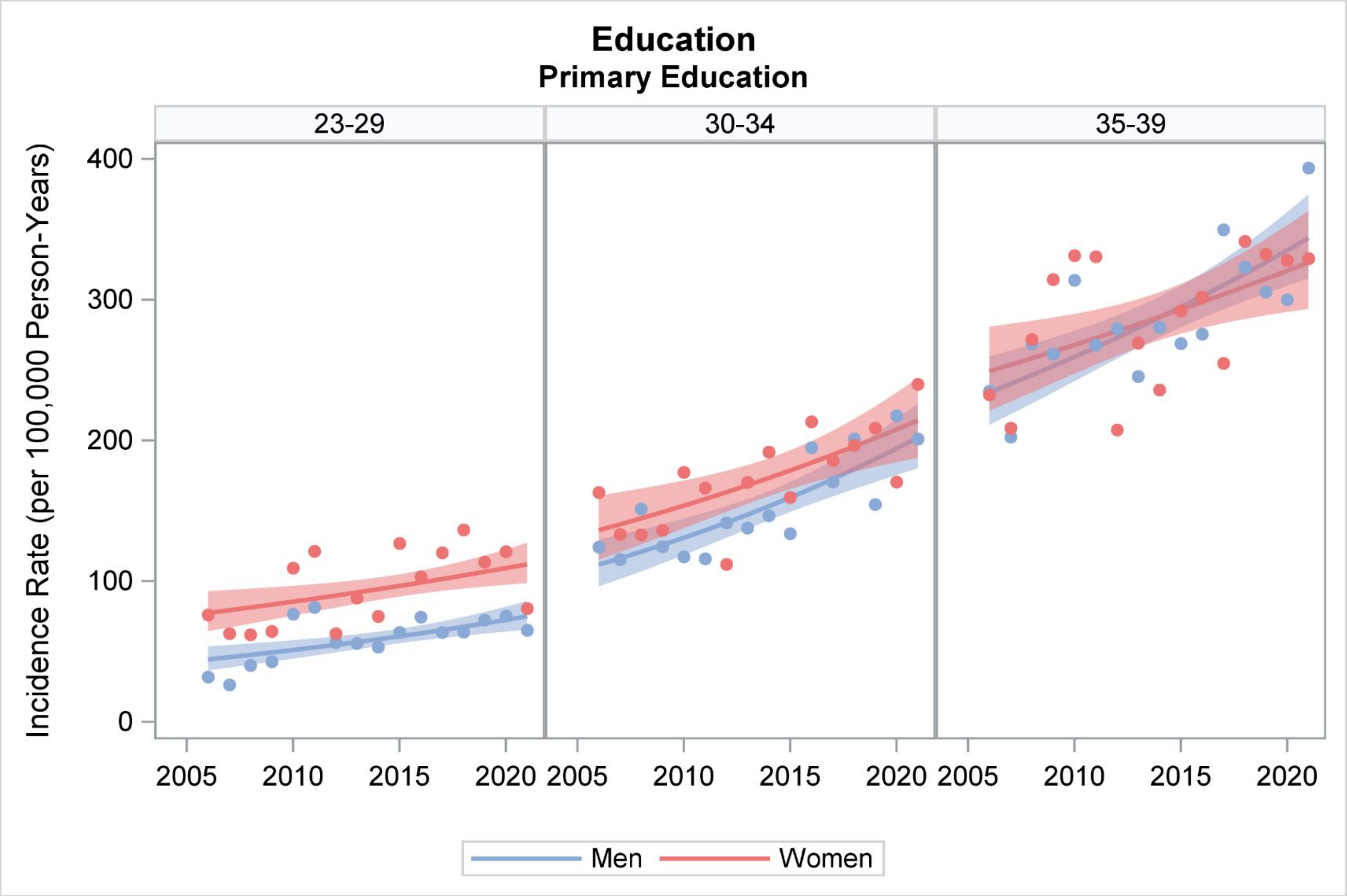

Figure S6. Incidence (per 100 000) with 95% confidence intervals of early-onset type 2 diabetes 2006 to 2021by sex in people with secondary education.

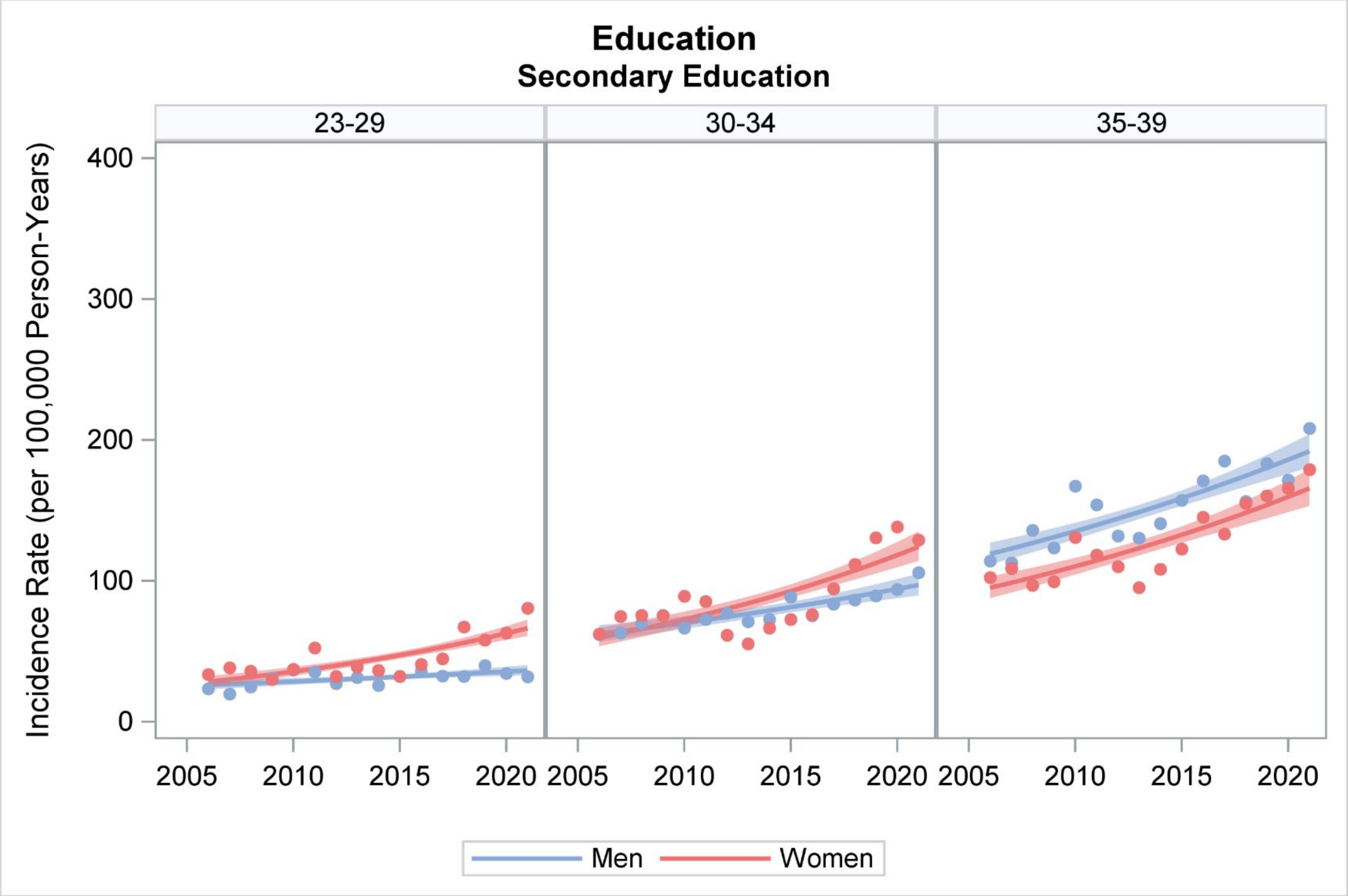

Figure S7. Incidence (per 100 000) with 95% confidence intervals of early-onset type 2 diabetes 2006 to 2021by sex in people with tertiary education.

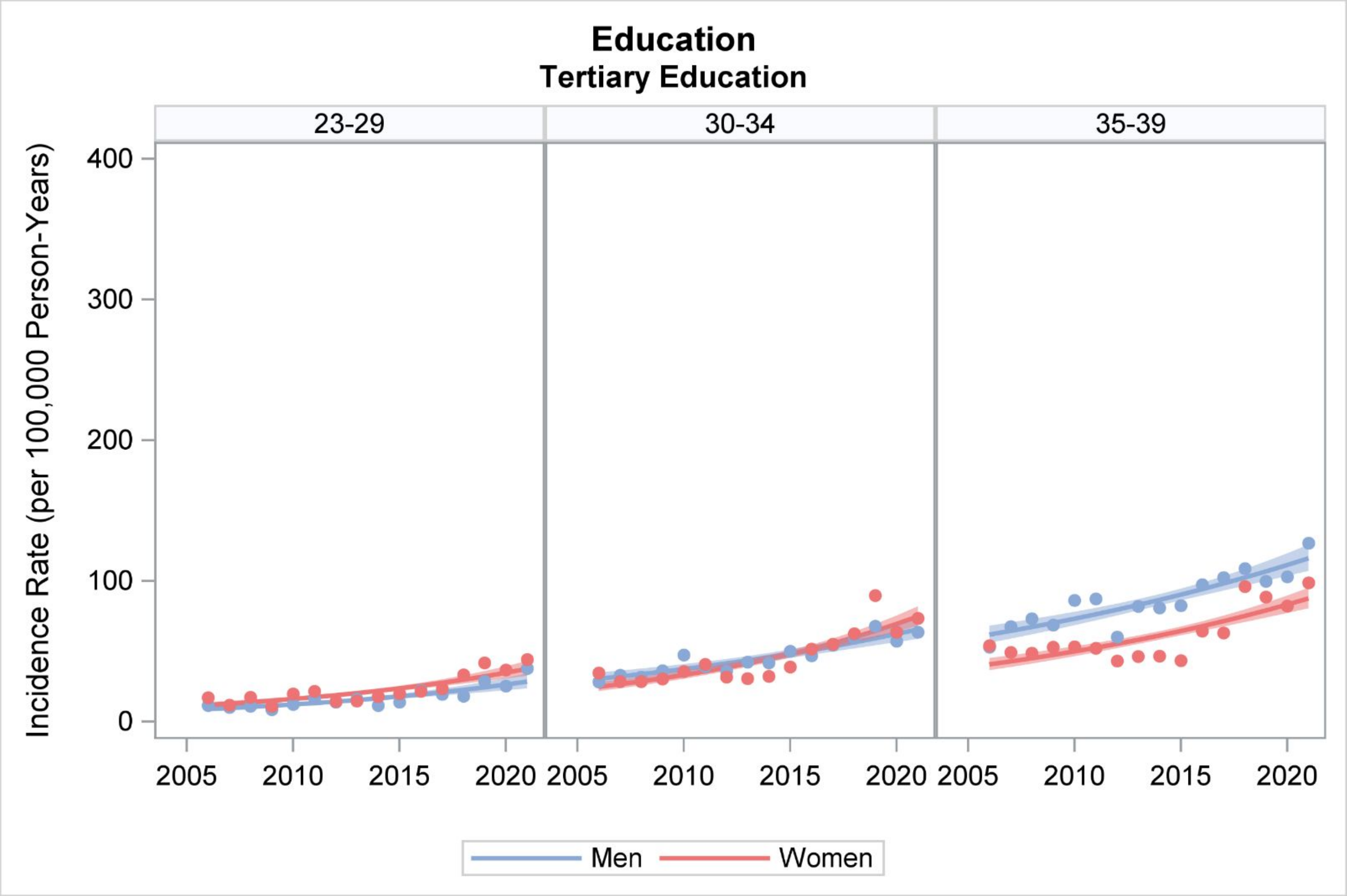


41

Table S12. Projected prevalence of type 2 diabetes in the age group 23-39 year, 2022 to 2050.

| Year | Men | Women | Total |
|---|---|---|---|
| **2021** | 0.58% | 0.69% | 0.64% |
| **2022** | 0.64% | 0.76% | 0.70% |
| **2023** | 0.70% | 0.83% | 0.76% |
| **2024** | 0.76% | 0.89% | 0.82% |
| **2025** | 0.80% | 0.95% | 0.87% |
| **2026** | 0.83% | 1.01% | 0.92% |
| **2027** | 0.89% | 1.08% | 0.98% |
| **2028** | 0.94% | 1.15% | 1.04% |
| **2029** | 0.99% | 1.22% | 1.10% |
| **2030** | 1.02% | 1.29% | 1.15% |
| **2031** | 1.05% | 1.35% | 1.19% |
| **2032** | 1.08% | 1.43% | 1.25% |
| **2033** | 1.12% | 1.52% | 1.31% |
| **2034** | 1.16% | 1.60% | 1.37% |
| **2035** | 1.20% | 1.70% | 1.44% |
| **2036** | 1.24% | 1.80% | 1.51% |
| **2037** | 1.29% | 1.91% | 1.59% |
| **2038** | 1.34% | 2.02% | 1.67% |
| **2039** | 1.38% | 2.14% | 1.75% |
| **2040** | 1.44% | 2.27% | 1.85% |
| **2041** | 1.50% | 2.42% | 1.95% |
| **2042** | 1.56% | 2.57% | 2.05% |
| **2043** | 1.63% | 2.74% | 2.17% |
| **2044** | 1.69% | 2.91% | 2.29% |
| **2045** | 1.77% | 3.10% | 2.42% |
| **2046** | 1.85% | 3.31% | 2.56% |
| **2047** | 1.94% | 3.54% | 2.72% |
| **2048** | 2.02% | 3.77% | 2.88% |
| **2049** | 2.11% | 4.03% | 3.04% |
| **2050** | 2.20% | 4.29% | 3.22% |

For Peer Review Only

**Table S13. Clinical characteristics of people with incident type 2 diabetes by age at diagnosis.**

| | Early-onset T2D, diagnosed at age 23-39 years | | | Late-onset T2D, diagnosed at age ≥40 years | | |
|---|---|---|---|---|---|---|
| | Men | Women | Total | Men | Women | Total |
| **No.** | 13 062 | 11 148 | 24 210 | 286 290 | 219 285 | 505 575 |
| **% Smokers** | 24.9% | 19.0% | 22.4% | 15.8% | 15.1% | 15.5% |
| **BMI (kg/m²)** | | | | | | |
| **Mean (SD)** | 33.9 (7.2) | 35.4 (7.8) | 34.5 (7.5) | 30.3 (5.2) | 31.1 (6.2) | 30.6 (5.7) |
| **% with obesity** | 69.2% | 75.2% | 71.6% | 47.9% | 53.8% | 50.3% |
| **HbA1c (mmol/mol)** | | | | | | |
| **Mean (SD)** | 63 (23) | 55 (19) | 59 (22) | 55 (19) | 53 (16) | 54 (18) |
| **% within target** | 43.7 | 60.0 | 50.3 | 62.3 | 68.4 | 64.9 |
| **Triglycerides mmol/L** | | | | | | |
| **Mean (SD)** | 2.9 (2.9) | 2.0 (1.7) | 2.6 (2.5) | 2.1 (1.7) | 1.9 (1.2) | 2.0 (1.5) |
| **% within target** | 33.2% | 49.4% | 39.5% | 48.8% | 50.2% | 49.4% |
| **Systolic blood pressure mmHg** | | | | | | |
| **Mean (SD)** | 130 (15) | 124 (15) | 128 (15) | 136 (17) | 137 (17) | 137 (17) |
| **% within target** | 72.9% | 84.4% | 77.5% | 56.6% | 55.55 | 56.1% |
| **eGFR mL/min/1.73m²** | | | | | | |
| **Mean (SD)** | 114.3 (25.8) | 111.8 (27.6) | 113.3 (26.6) | 87.2 (22.9) | 81.1 (23.6) | 84.6 (23.4) |
| **% within target** | 99.6% | 99.3% | 99.5% | 90.8% | 83.6% | 87.7% |
| **% with Albuminuria** | 17.6% | 15.6% | 16.9% | 18.2% | 13.5% | 16.2% |
| | | | | | | |

Being within target was defined as having HbA1c <7% (53 mmol/mol)[1], systolic blood pressure <140 mmHg[2], triglycerides <1.7 mmol/L[3], and eGFR ≥60 mL/min/1.73 m². Obesity was defined as BMI ≥30 and Albuminuria as micro or macro albuminuria. The clinical information was obtained from the Diabetes Register for the year of diagnosis or the earliest recording thereafter.

**Table S14. Clinical characteristics of people with incident early-onset type 2 diabetes by age at diagnosis.**

| | Men | | | Women | | | Total | | |
|---|---|---|---|---|---|---|---|---|---|
| **Age** | 23-29 | 30-34 | 35-39 | 23-29 | 30-34 | 35-39 | 23-29 | 30-34 | 35-39 |
| **n** | 2 217 | 3 806 | 7 039 | 2 703 | 3 487 | 4 958 | 4 920 | 7 293 | 11 997 |
| **% Smokers** | 24.4 | 24.2 | 25.3 | 22.0 | 18.0 | 18.3 | 23.3 | 21.6 | 22.6 |
| **BMI (kg/m²)** | | | | | | | | | |
| **mean (SD)** | 35.6 (7.8) | 34.3 (7.3) | 33.3 (6.8) | 36.5 (8.2) | 35.6 (7.8) | 34.9 (7.5) | 36.0 (8.0) | 34.8 (7.6) | 33.9 (7.1) |
| **% with obesity** | 76.6 | 70.1 | 66.6 | 78.8 | 76.2 | 73.3 | 77.6 | 72.6 | 69.2 |
| **HbA1c mmol/mol** | | | | | | | | | |
| **mean (SD)** | 63 (23) | 63 (23) | 63 (23) | 56 (20) | 54 (19) | 54 (18) | 60 (22) | 59 (22) | 59 (22) |
| **% within target** | 41.0 | 42.5 | 45.0 | 56.5 | 61.4 | 60.5 | 48.1 | 50.3 | 51.0 |
| **eGFR mL/min/1.73m²** | | | | | | | | | |
| **mean (SD)** | 124.2 (27.0) | 116.6 (26.4) | 110.4 (24.3) | 119.3 (29.1) | 113.2 (27.5) | 108.1 (26.4) | 121.9 (28.1) | 115.2 (26.9) | 109.5 (25.2) |
| **% within target** | 99.8 | 99.6 | 99.5 | 99.6 | 99.5 | 99.1 | 99.7 | 99.6 | 99.4 |
| **Triglycerides mmol/L** | | | | | | | | | |
| **mean (SD)** | 3.0 (2.9) | 3.0 (3.1) | 2.9 (2.9) | 2.2 (2.1) | 2.0 (1.4) | 2.0 (1.6) | 2.6 (2.6) | 2.6 (2.6) | 2.5 (2.5) |
| **% within target** | 30.8 | 33.3 | 33.7 | 44.4 | 50.1 | 50.8 | 36.8 | 39.8 | 40.2 |
| **Systolic blood pressure mmHg** | | | | | | | | | |
| **mean (SD)** | 130 (14) | 130 (15) | 131 (15) | 122 (13) | 123 (15) | 125 (15) | 126 (14) | 127 (15) | 128 (15) |
| **% within target** | 73.7 | 74.9 | 71.7 | 87.2 | 85.7 | 82.6 | 79.9 | 79.3 | 75.9 |
| **% with Albuminuria** | 18.7 | 17.5 | 17.6 | 15.6 | 14.6 | 16.3 | 17.3 | 16.3 | 17 |

Being within target was defined as having HbA1c <7% (53 mmol/mol)[1], systolic blood pressure <140 mmHg[2], triglycerides <1.7 mmol/L[3], and eGFR ≥60 mL/min/1.73 m². Obesity was defined as BMI ≥30 and Albuminuria as micro or macro albuminuria. The clinical information was obtained from the Diabetes Register for the year of diagnosis or the earliest recording thereafter. This information was available for 70.1% of the patients.

For Review Only

References

1. Holt RIG, DeVries JH, Hess-Fischl A, et al. The Management of Type 1 Diabetes in Adults. A Consensus Report by the American Diabetes Association (ADA) and the European Association for the Study of Diabetes (EASD). *Diabetes Care* 2021;44:2589-2625. DOI: 10.2337/dci21-0043.

2. Rawshani A, Rawshani A, Franzén S, et al. Risk Factors, Mortality, and Cardiovascular Outcomes in Patients with Type 2 Diabetes. *N Engl J Med* 2018;379:633-644. DOI: 10.1056/NEJMoa1800256.

3. Standards of Medical Care in Diabetes-2022 Abridged for Primary Care Providers. *Clin Diabetes* 2022;40:10-38. DOI: 10.2337/cd22-as01.

For Review Only

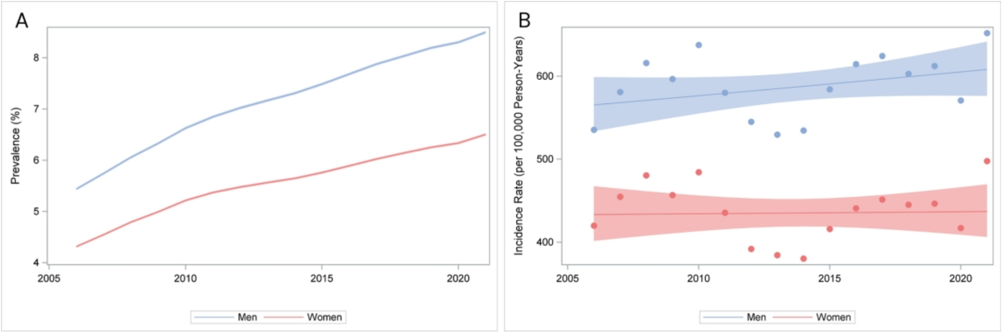


Figure 1A. Age-standardized prevalence (%) of type 2 diabetes in Sweden 2006-2021 by sex. The shaded areas represent 95% confidence intervals.
Figure 1B. Age-standardized incidence (per 100 000 person-years) of type 2 diabetes in Sweden 2006-2021 by sex. The shaded areas represent 95% confidence intervals

175x57mm (300 x 300 DPI)

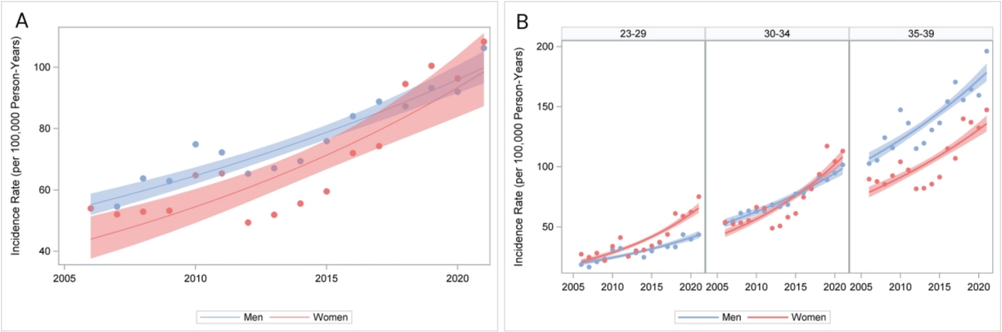


Figure 2A. Age-standardized incidence (per 100 000 person-years) of early-onset T2D in Sweden 2006-2021 by sex. Annual change: 4.7% (CI 3.7-5.7%) overall, 4.0% (CI 3.4—4.7%) in men, 5.5% (CI 3.8—7.2%) in women. The shaded areas represent 95% confidence intervals (CI).
Figure 2B. Incidence (per 100 000 person-years) of early-onset T2D in Sweden 2006-2021 by age and sex. Annual change: Age 23-29; 6.7% (CI 6.0—7.4%) overall, 5.3% (CI 4.4—6.4%) in men, 7.9% (CI 3.8—7.2%) in women, Age 30-34; 5.1% (CI 4.6—5.6%) overall, 4.2% (CI 3.5—4.9%) in men, 6.1% (CI 6.9—8.8%) in women, Age 35-39; 3.6% (CI 3.2—4.0%) overall, 3.5% (CI 3.0—4.4%) in men, 3.7% (CI 3.1—4.9%) in women. The shaded areas represent 95% confidence intervals (CI).

175x57mm (300 x 300 DPI)

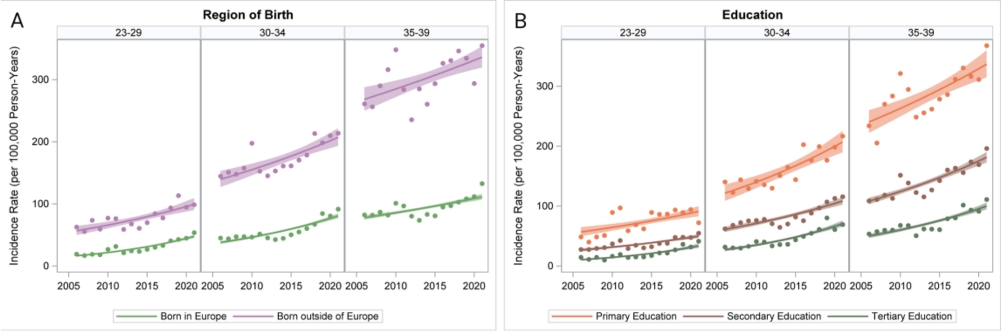


Figure 3A. Incidence (per 100 000 person-years) of early-onset T2D 2006-2021 by age and region of birth. The shaded areas represent 95% confidence intervals. Annual change; in people born in Europe: Age 23-29; 7.4% (CI 6.6—8.2%) Age 30-34; 5.2% (CI 4.5—5.8%), Age 35-39; 2.4% (CI 1.9—2.9%). In people born outside Europe: Age 23-29, 3.8% (CI 2.5—5.2%), Age 30-34, 3.8% (CI 2.5—5.2%), Age 35-39, 1.5% (CI 0.8—2.2%).

Figure 3B. Incidence (per 100 000 person-years) of early-onset T2D 2006-2021 by age and highest attained education. The shaded areas represent 95% confidence intervals. Annual change; in people with primary education: Age 23-29; 3.2% (CI 1.9—4.5%) Age 30-34; 3.6% (CI 2.5—4.8%), Age 35-39; 2.3% (CI 1.4—3.1%). In people with secondary education: Age 23-29, 4.3% (CI 3.3—5.2%), Age 30-34, 3.9% (CI 3.1—4.7%), Age 35-39, 3.5% (CI 2.9—4.1%). In people with tertiary education: Age 23-29, 8.1% (CI 6.6—9.6%), Age 30-34, 6.6% (CI 5.6—7.6%), Age 35-39, 4.7% (CI 3.9—5.5%)

175x57mm (300 x 300 DPI)

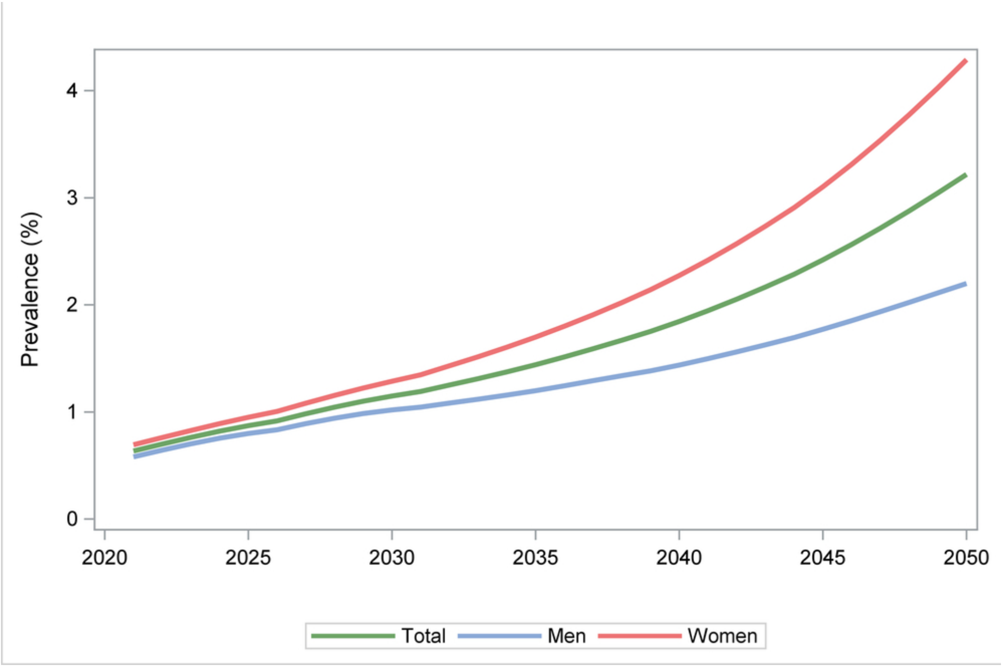


Figure 4. Projected prevalence (%) of type 2 diabetes in the age group 23-39 years, 2022 to 2050.

84x55mm (300 x 300 DPI)
